# Deriving High-Resolution Protein Backbone Structure Propensities from All Crystal Data Using the Information Maximization Device

**DOI:** 10.1371/journal.pone.0094334

**Published:** 2014-06-04

**Authors:** Armando D. Solis

**Affiliations:** Biological Sciences Department, New York City College of Technology, The City University of New York, Brooklyn, New York, United States of America; University of Queensland, Australia

## Abstract

The most informative probability distribution functions (PDFs) describing the Ramachandran phi-psi dihedral angle pair, a fundamental descriptor of backbone conformation of protein molecules, are derived from high-resolution X-ray crystal structures using an information-theoretic approach. The Information Maximization Device (IMD) is established, based on fundamental information-theoretic concepts, and then applied specifically to derive highly resolved phi-psi maps for all 20 single amino acid and all 8000 triplet sequences at an optimal resolution determined by the volume of current data. The paper shows that utilizing the latent information contained in all viable high-resolution crystal structures found in the Protein Data Bank (PDB), totaling more than 77,000 chains, permits the derivation of a large number of optimized sequence-dependent PDFs. This work demonstrates the effectiveness of the IMD and the superiority of the resulting PDFs by extensive fold recognition experiments and rigorous comparisons with previously published triplet PDFs. Because it automatically optimizes PDFs, IMD results in improved performance of knowledge-based potentials, which rely on such PDFs. Furthermore, it provides an easy computational recipe for empirically deriving other kinds of sequence-dependent structural PDFs with greater detail and precision. The high-resolution phi-psi maps derived in this work are available for download.

## Introduction

Accurate descriptions of the natural propensities of backbone conformation of proteins serve to improve the analysis and prediction of protein structures. The structure of the protein backbone is frequently characterized by a series of dihedral angle pairs, the phi-psi angles, defined by the rotation around two bonds: one bond between the alpha carbon and the amine marks the phi angle, while the other bond between the alpha carbon and the carbonyl marks the psi angle. The Ramachandran phi-psi space [1], defined by these two torsions, has provided an indispensable summative description of backbone conformation. The ubiquitous Ramachandran plots, which recently celebrated their 50^th^ anniversary [2], have become an insightful and widely accepted illustration of key properties of protein secondary structure as well as a powerful tool for molecular structure analysis [3].

Analyzing databases of experimentally derived molecular structures of proteins has long served as a powerful way to explore allowed and disallowed regions of the phi-psi space [4], [5]. Mapping this space empirically is straightforward in principle: plots are assembled as a distribution of observed frequencies, culled from a statistical survey of high-resolution structures. Frequency plots from a considerably large database of structures, representing the range of known conformations, serve as estimates of structural propensities or probabilities. With sufficient data, amino-acid specific plots can be assembled easily. Such probability density or distribution functions (PDFs) demonstrate sequence dependence of backbone conformation in folded protein environments, and may suggest key local effects in protein folding [6].

High-resolution phi-psi maps have been useful in important applications including structure validation [7]–[9] and structure refinement [10], [11], and have provided insight into the nuances of sequence-dependent protein structure [12]–[14]. Naturally, these PDFs have been integrated into so-called “knowledge-based” potential functions (KBPs) used for protein structure prediction [15]–[18]. Over time, these PDFs have been refined as the repository of structural data, the Protein Data Bank (PDB), grew and as the proportion of high-resolution X-ray structures increased. In the past decade, the explosion in the number of viable structures and the concomitant increase in the number of known protein folds have prompted fresh insights into the sequence dependence of backbone conformation [3], [19].

It should come as no surprise that empirical PDFs (and their associated knowledge-based potentials) are acutely dependent on the data set from which they are derived. Two prescriptions have normally guided the organization of such data sets. First, data sets aim to be *comprehensive*. They seek to include all the known folds as well as the rich variety of amino acid sequences. Maximizing both the number of structures and the coverage across sequence and fold spaces should result in more accurate probability estimates, yielding as much detail as current data can provide. Second, data sets aim to be *non-redundant*. Because some protein folds and even some individual proteins are overrepresented in the PDB, a selection must be made to include only a fair number of each fold and sequence in order to avoid bias. The underlying rationale for these two prescriptions is to recover some measure of the intrinsic structural propensities. These considerations have given rise to automatically-generated, comprehensive, non-redundant sets such as PDB-SELECT [20] and PISCES [21], which are updated periodically to keep up with the expansion of the universe of solved protein structures. These data sets have been used to derive many knowledge-based potential functions, from local backbone torsions to long-range side-chain contacts. While common practices regarding the construction of data sets have advanced the field significantly, I demonstrate later in this paper that additional refinements in the method of constructing data sets can yield significantly improved performance.

After the assembly of a coherent structural data set, the next important consideration is how exactly PDFs will be computed. Compromises have to be made in the descriptors employed when assembling any type of sequence-dependent structural PDF because data are limited. The number of unique amino acid fragments explodes exponentially as longer polypeptide sequences are considered; therefore, any study of the sequence dependence of backbone conformation is restricted to the shortest length scales. Moreover, the resolution of the resulting empirical phi-psi maps is bound to be limited since the data are subdivided among however many short sequences there are, reducing the number of occurrences per subdivision. Some approaches to deriving KBPs employ sparse data corrections to address this problem. Among them, the Sippl information quantum [22], an arbitrary constant, is the most widely used. This paper argues for a more systematic derivation of descriptors.

Persistent questions about how data sets are to be assembled, along with the actual process of estimating probabilities from such data sets, therefore form the impetus for the current work. To date, not much has been done to closely examine the effect of decisions made regarding these issues, even though the processes of data selection and probability estimation are as ubiquitous as KBPs themselves. In this work, I outline a theoretical framework to investigate the relevant probabilities directly and develop a tool, the Information Maximization Device (IMD), that can be applied to a range of computational problems. This tool has allowed me to exploit empirical data in deriving the most accurate high-resolution phi-psi angle pair PDFs for all 20 amino acids and all 8000 full-sequence triplets to date.

Aside from the ability to derive information-rich PDFs, another primary benefit of using an information-theoretic approach rests on the connection we established [23], [24] between increased mutual information estimates given by the PDFs and improved performance of KBPs in protein structure prediction, using threading as a model. Due to this fundamental relation, estimating the information content of any derived set of PDFs becomes a more meaningful endeavor. Since information can indicate the efficiency by which empirical data is being used, variations in the way data sets are organized can be tested and compared. In such cases, maximizing information becomes an appropriate objective function, in that it facilitates the selection of the most effective structural PDFs.

There have been a number of efforts to formulate backbone dihedral angle PDFs from statistical surveys of structural data throughout the years. The most basic structural maps chart the natural propensities for each of the 20 amino acids [9], [12], [25]. More recently, maps incorporating nearest neighbor residues have been elucidated as well—for dipeptides [26] and also for tripeptides, both with reduced [23], [27] and the full 20-letter alphabets (by Betancourt [16]). The challenge in establishing numerous near-neighbor PDFs is the sparseness of available data per sequence. The work described here deals directly with this challenge, and suggests an approach that yields tripeptide PDFs of greater accuracy. In exploring guidelines for formulating the data sets that yield information-rich PDFs, I demonstrate that the most informative PDFs are those that are derived from *all* high-resolution crystal data. As a display of their utility, I show here these PDFs perform significantly better than Betancourt's tripeptide PDFs in fold recognition.

Our previous work [23] used a similar but less refined strategy to explore the local sequence dependence of the phi-psi dihedral angles. This paper continues that work and makes significant progress: specifically, here I advance (1) a better articulation of the probability estimation method, which is here fully integrated into the Information Maximization Device, (2) a theoretical rationale for distinguishing between the data sets involved in probability estimation and evaluating their effectiveness, (3) a recipe for using all high-resolution X-ray structures currently in the PDB, which greatly expands the ability to estimate PDFs, (4) a way to more efficiently discretize structural space, which permits the highest possible resolution. Together these outcomes yield (5) a full elucidation of the high-resolution phi-psi maps of the 20 amino acids and the central residues of all 8000 triplet amino acid sequences. The PDFs for these phi-psi maps are available for download.

## Materials and Methods

The relationship between amino acid sequence and its native molecular structure can be explored in a variety of ways, from approaches based on biophysical notions to bioinformatics methodologies relying on probabilistic models. Our previous work [23], [24], [27] employed information theory to help resolve the protein sequence-structure relationship. Information theory is a natural framework with which to analyze this relationship, due to a number of reasons that I shall elucidate throughout this paper. Foremost among these is Anfinsen's dogma [28], the fundamental concept of folding, which declares that the information needed to fold a (globular) protein into its three-dimensional conformation lies completely in its sequence of amino acids. Besides posing an elegant biophysical puzzle, this dogma raises an obvious question for information theory. Mathematically, the operative probability distribution function *p*(*C*|*S*), where *C* is conformation and *S* is sequence, naturally represents the relationship between sequence and structure, and lends itself to the basic equations of information theory as well as of Boltzmann energetics.

This section is organized as follows. Section A summarizes the relationship between knowledge-based potentials (KBPs) and mutual information, and in particular the ability to optimize the performance of KBPs by maximizing mutual information. Section B distinguishes between two kinds of sequence-dependent structural probabilities that are at play in KBPs—the true underlying probability and our best estimate for it, derived using empirical (finite) data. An information-theoretic connection between these two probability distributions sets the stage for a simple computational procedure to derive the best estimate for the true underlying probability, the Information Maximization Device (IMD), as discussed in Section C. Section D discusses particular requirements for operating the IMD, and Section E describes the data sets and specific procedures used in this work in the application of the IMD to derive the best-performing local-sequence-dependent phi-psi maps.

### A. Fundamental connection: Mutual Information and Potentials of Mean Force

The connection between information-theoretic quantities and statistical/knowledge-based potentials (KBPs) is established by invoking the concept of mutual information, *I*(*C*,*S*), which measures the information overlap between conformation *C* and sequence *S* [23]. Specifically, the discrete form of the information equation is as follows:
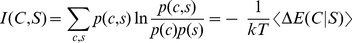
(1)where *k* is the Boltzmann constant, *T* is the absolute temperature, lower cases (*c* and *s*) represent specific instances, and *p* represents probabilities as they exist in nature. The summations run through the set of all sequences and conformations that exist in the universe of native folds. The energy function Δ*E* arises from the Boltzmann formalism, a fixture of many knowledge-based potentials [29], whose expected value we showed previously to be equivalent to mutual information [23]. Such an equivalence provides the basis for considering energetic quantities purely on an information-theoretic framework; that is, using mean-force energies such Eq.(1) in structure prediction is effective precisely because they are also information-theoretic quantities, whose significant advantage lies in being free from stringent physics-based considerations. Using information-theoretic terms as an objective function instead of energies grants the freedom to manipulate parameters and functionalities without need for physical justification [23].

One challenge in computing the quantities in the equation above is how to adequately estimate probabilities from frequency counts in a finite structural database, given that the resolution of the structural descriptor is also variable. For instance, the phi-psi space may be discretized into any resolution desired, but the pressure of sparse data at high resolutions may render such probabilities ultimately uninformative. A rational way of deciding these issues is to apply an information maximization principle to Eq.(1). Specifically, because manipulating structural and sequence parameters that define *C* and *S*, as well as the functionality of *p*, will affect the value of mutual information, the most optimal parameterization and functionality are those that maximize mutual information. A search through sequence and structural descriptors for local backbone and long-range contact interactions has revealed physically consistent patterns that have proven especially robust in comparison to other approaches [23], [24]. The same principle will be applied in this work to search for an efficient method to derive probabilities empirically, and in the process identify the best way to utilize as much latent information as exists in the set of high-resolution structures in the Protein Data Bank (PDB).

The underlying power of the principle of information maximization rests on the observation that knowledge-based potentials built from highly informative PDFs perform best in structure prediction tests [23], [24]. Therefore, parameters and procedures that have been formulated by maximizing information should assist in improving the performance of potentials. The prescription is simple: any variable or procedure involved in the estimation of PDFs used for KBPs can be optimized by maximizing information. The variables that can be optimized include sequence descriptors (e.g., number of amino acids considered, reduction of amino acid alphabet) and structure descriptors (e.g., resolution). In this work, I apply this optimization principle to two fundamental, underlying aspects of KBPs: (1) the method of estimating discrete probabilities and (2) the assembly of data sets used to estimate these probabilities. In addition, I formulate a straightforward computational device to implement such an optimization.

### B. The basis of information maximization: reducing the difference between empirically estimated probabilities and true underlying probabilities

This section establishes the basis for accurately estimating underlying sequence-dependent structural probabilities using experimental structure data, for use in the formulation of knowledge-based potentials (KBPs). The key is to explore the relationship between unknown true underlying probabilities and empirically estimated probabilities by using fundamental information-theoretic measures. Probabilities are converted into KBPs via the Boltzmann principle (routinely simplified by dropping the partition function term), which links equilibrium probabilities of backbone conformational states with energy Δ*E* [29]:
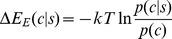
(2)where *s* and *c* are particular cases of sequence and conformation respectively. The two PDFs, *p*(*c*|*s*) and *p*(*c*), are the components of the KBP that reflect structural propensities in natural proteins: *p*(*c*|*s*) is the sequence-dependent probability, while *p*(*c*) is the sequence-independent structural probability, also called the reference state, which can be derived directly from *p*(*c*|*s*). The subscript “E” on Δ*E* emphasizes the fact that it is not necessarily an energy in the classical sense, but an empirical quantity dependent on how *p* is defined.

A crucial distinction between two probability distributions has to be made at the outset: there exists a true underlying probability distribution *p_T_*(*c|s*) which operates in nature but is unknown, and there is *p_E_*(*c|s*), an estimate for the true distribution, derived from empirical data. The difference between the estimate *p_E_*(*c|s*), and the true *p_T_*(*c|s*) can be measured by the distance between these two PDFs, as expressed by the information-theoretic quantity Kullback-Leibler divergence [30]:
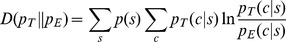
(3a)which can be expanded thus:
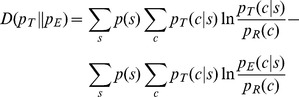
(3b)


The Gibbs' inequality, a fundamental property of divergence, states that *D*≥0 [30], transforming this equation into the following:
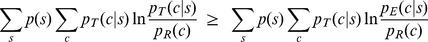
(4a)


The left-hand side of the inequality is the true mutual information between the sequence *S* and the structure *C* domains, or *I*(*C*,*S*), as described in the Eq.(1). This level of mutual information is achieved only when the true underlying probabilities *p_T_*(*c*|*s*) are known completely. The right-hand side can be viewed as an estimate of the mutual information, indicated by *I_E_*(*C*,*S*). This quantity is what is computed using *p_E_*(*c*|*s*), estimated empirically from currently known data. The inequality can be simplified thus:
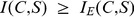
(4b)


This formulation results in the following chain of consequences. First, any estimate for mutual information computed from finite data will be less than the true mutual information. Equality is only possible when *p_T_*(*c*|*s*) = *p_E_*(*c*|*s*), the point at which the empirical probabilities perfectly estimate the true underlying probabilities. The inequality above suggests that the divergence or “distance” between the estimate *p_E_*(*c*|*s*) and the underlying true *p_T_*(*c*|*s*) can be measured by the difference between *I_E_*(*C*,*S*) and *I*(*C*,*S*). Thus, within the realm of finite data, as the *p_E_*(*c*|*s*) estimate for *p_T_*(*c*|*s*) improves, the mutual information estimate *I_E_*(*C*,*S*) approaches the true mutual information *I*(*C*,*S*). Therefore, better probability estimation—by the efficient use of available data and by employing more effective ways of transforming observed frequencies into probabilities—should assist in increasing the value of the mutual information estimate.

It follows that the resulting *p_E_*(*c*|*s*) that yields the maximum mutual information estimate, max{*I_E_*(*C*,*S*)}, is the best estimate for *p_T_*(*c*|*s*). This implies that an information maximization strategy should naturally produce the best PDFs that the data allow. It should not be surprising that the increased accuracy in estimating *p_E_*(*c*|*s*) due to information maximization also benefits the quality of the KBP, as gauged by its performance in fold recognition [23], [24]. These interrelated ideas form the basis for a rational, information-based approach to problems concerning limited structural data and database assembly.

### C. The Information Maximization Device: computing and maximizing mutual information

The mutual information estimate *I_E_*(*C*,*S*) derived in the section above explicitly includes *p_T_*(*c*|*s*), the *unknown* quantity that we are trying to estimate via the information maximization principle. Basic ideas about expectation suggest how to compute mutual information in practical applications. The double summation applied across all native sequences *s* and all native structures *c* on the right hand side of Eq.(4a) can be recast as an expectation of the log-odds score across the universe of native sequences and structures:
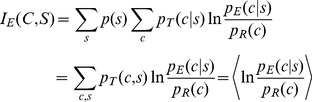
(5a)which can be estimated as:
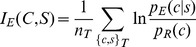
(5b)using a data set {*c*,*s*}_T_ that is sufficiently large and representative of the diversity of sequences and conformations in the universe. Recasting the mutual information estimate this way permits its evaluation while bypassing *p_T_*(*c*|*s*), which is unknown.

The equation above reveals a simple recipe: *p_E_*(*c*|*s*) and *p_R_*(*c*) are the components of the potential function, and the log of their ratio is related to the “energy” of the particular (*c*,*s*) combination as well as its contribution to the overall *I_E_*(*C*,*S*). The summation is applied to a range of native (*c*,*s*) pairs found in the data set {*c*,*s*}_T_ composed of *n_T_* protein chains, resulting in the mean energy and also *I_E_*(*C*,*S*). Varying a multitude of conditions, including how *p_E_*(*c*|*s*) is estimated from empirical data and also the resolutions of both sequence and conformation, will change *I_E_*(*C*,*S*). A rigorous search through these different conditions yields the *best estimate* for *p_T_*(*c*|*s*): it is the *p_E_*(*c*|*s*) which carries the maximum mutual information estimate:
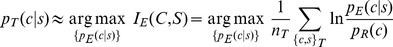
(6)


The expression above generalizes the Information Maximization Device. For its proper computation, it needs two main ingredients: (1) a comprehensive data set {*c*,*s*}_T_ covering a diversity of sequences and conformations, and; (2) a method to generate empirical probability estimates *p_E_*(*c*|*s*) and *p_R_*(*c*) (the components of the KBP)_._

The optimal probability estimate *p_E_*(*c*|*s*) can be generated using any method, including brute force Monte Carlo-type approaches if the number of states is small. To construct estimates for complex probability distributions with many states, one can employ methods to estimate probabilities from an empirical data set {*c*,*s*}_E_, which is distinct from {*c*,*s*}_T_. In order for IMD to avoid data over-fitting, the data set {*c*,*s*}_E_, which can be thought of as the training set, must be non-overlapping with {*c*,*s*}_T_, which can be thought of as the testing set. At the minimum, applying jackknifing strategies should ensure that the two data sets do not overlap. The goal is to ensure that when computing each term of the summation in Eq.(6), which represents one particular sequence and its native conformation, the data set {*c*,*s*}_E_ used to estimate *p_E_*(*c*|*s*) does not contain that sequence.

**Figure 1:**
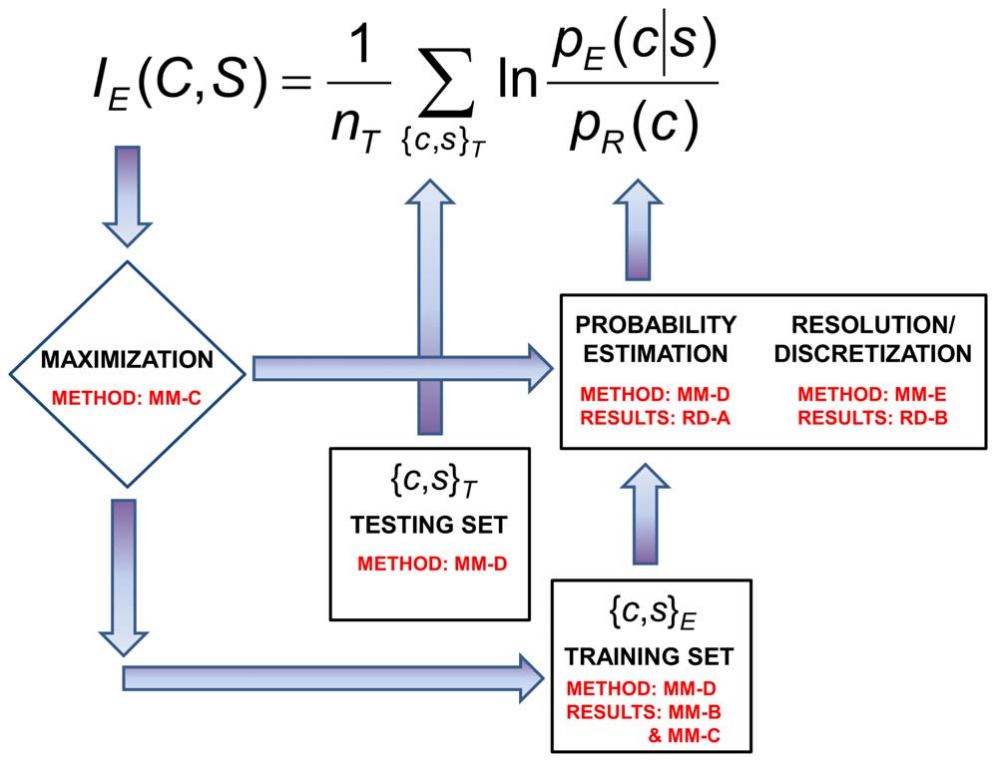
The Information Maximization Device (IMD). The ingredients of the iterative optimization procedure are illustrated, and relevant Methods and Results sections are indicated. The objective function for the IMD optimization is the mutual information *I_E_*(*C*,*S*). The quantity *n_T_* is the number of data points (or amino acid residues) in the testing set, *p_E_*(*c*|*s*) is the PDF being estimated, and *p_R_*(*c*) is the reference state, in this case the sequence-independent phi-psi dihedral angle pair PDF. Two data sets are necessary for the procedure—the training set to assemble PDF estimates and the testing set to subject these PDFs to mutual information measurement. The training set can be treated as a variable; in this work for instance, different data sets PDBSEL and BLCLUST are evaluated for their effectiveness in probability estimation. On the other hand, the testing set, carefully assembled to reflect the system for which the PDFs are being estimated, is treated as a constant. Here, the probability estimation procedure is also a variable to be optimized, as well as other factors relevant to constructing PDFs, including resolution and the specific partitioning of the structural space. Searching through the variable space for the highest mutual information yields the best set of PDFs.

Figure 1 gives a graphic illustration of the IMD, including requirements concerning data as well as the procedures of probability estimation and structural discretization that are required for iterative information-based optimization. The figure also refers to the individual methods employed for the specific purpose of formulating high-resolution phi-psi plots, and the results pertaining to each aspect of the methodology.

### D. Implications and General Procedures

The formulations above suggest a cogent way to derive the most accurate PDFs from empirical data. The specific tasks for deriving the best estimate for *p_T_*(*c*|*s*) are: (1) assembling two comprehensive data sets {*c*,*s*}_T_ and {*c*,*s*}_E_, the testing and training sets respectively; (2) deriving *p_E_*(*c*|*s*) and *p_R_*(*c*) from {*c*,*s*}_E_; and then (3) computing the mutual information estimate *I_E_*(*C*,*S*) using the IMD, Eq.(6). Manipulating the composition of data sets, probability estimation methods, and associated parameters alters *I_E_*(*C*,*S*); out of all possible scenarios, the *p_E_*(*c*|*s*) that maximizes *I_E_*(*C*,*S*) is the best estimate for *p_T_*(*c*|*s*). The procedures to accomplish these tasks are outlined below.

#### Assembling the testing set {*c,s*}_T_

In order to obtain the best approximations for the true underlying propensities, the testing set must be composed of a fair distribution of sequences and conformations reflecting the diversity of the universe of protein structures. More generally, the testing set {*c*,*s*}_T_ must embody the full range of sequences and conformations on which the KBP will be applied. For instance, KBPs specific to a subset of proteins (e.g. intrinsically disordered proteins, membrane proteins, etc.) ought to utilize a testing set that reflects this specificity.

#### Assembling the training set {*c*,*s*}_E_

The training set {*c*,*s*}_E_ used to estimate *p_E_*(*c*|*s*) and *p_R_*(*c*) is typically built from a diversity of natively folded chains. In many KBP studies, {*c*,*s*}_E_ is assembled from subsets of {*c*,*s*}_T_, frequently employing a simple jackknife method. This method involves removing one chain (*c*,*s*)*_i_* from the set {*c*,*s*}_T_, and then using the remaining set (i.e., {*c*,*s*}_T_ - (*c*,*s*)*_i_*) to predict the structure or to compute parameters relevant to that chain (*c*,*s*)*_i_*. This is repeated *n_T_* times, and an average quantity (either prediction accuracy or some other parameter) is computed. In this study, we are interested in computing the log-odds ratio between *p_E_*(*c*|*s*) and *p_R_*(*c*) for chain (*c*,*s*)*_i_* (Eq.(6)) using the data set {*c*,*s*}_E_ = {*c*,*s*}_T_-(*c*,*s*)*_i_* and then taking the mean across all chains in the testing set {*c*,*s*}_T_. The jackknife method prevents model over-fitting in limited data conditions, while still presenting a statistically robust way to estimate any parameter mean, including mutual information.

However, using ({*c*,*s*}_T_ - (*c*,*s*)*_i_*) is but one way to assemble the data set {*c*,*s*}_E_. In principle *p_E_*(*c*|*s*) and *p_R_*(*c*) can be derived by any mathematical or computational method; in fact, empirical estimates from frequency data need not be the only approach. The strength of the information-based approach lies in its ability to evaluate the viability of *any* given *p_E_*(*c*|*s*) and *p_R_*(*c*) however they were derived. The information maximization principle dictates that among PDFs generated by different approaches, those that generate the highest *I_E_*(*C*,*S*) are the best approximations for the underlying PDFs, regardless of how they were formulated. As a demonstration, this work describes an alternative way to assemble {*c*,*s*}_E_ by using *all* high resolution protein structures in the PDB, an approach that demonstrably yields more accurate PDFs.

#### Estimating *p_E_*(*c*|*s*) and *p_R_*(*c*)

A method for probability estimation must take into account size limitations of the data set, particularly when fine structural resolutions are used. The structural partition has to be of the right resolution to protect against data over-fitting, and the volume of data ought to dictate the optimal granularity of all descriptors.

One solution is to buttress sparsely populated frequencies with related well-defined frequency distributions. This involves using well-populated PDFs as a first approximation to boost poorly populated frequency distributions [16], [22], [23]. For instance, in estimating the PDF for the central dihedral angle pair of a rare triplet like Lys-Tyr-Gly from finite data, the raw frequencies for the single residue Tyr, which has significantly more occurrences in the data set, is used as the first approximation. A hybrid coefficient (analogous to Sippl's information quantum [22]) can be used to blend the raw triplet frequency with that of the single residue approximation. Such a coefficient, like any other adjustable parameter, can be optimized for mutual information as well.

**Figure 2:**
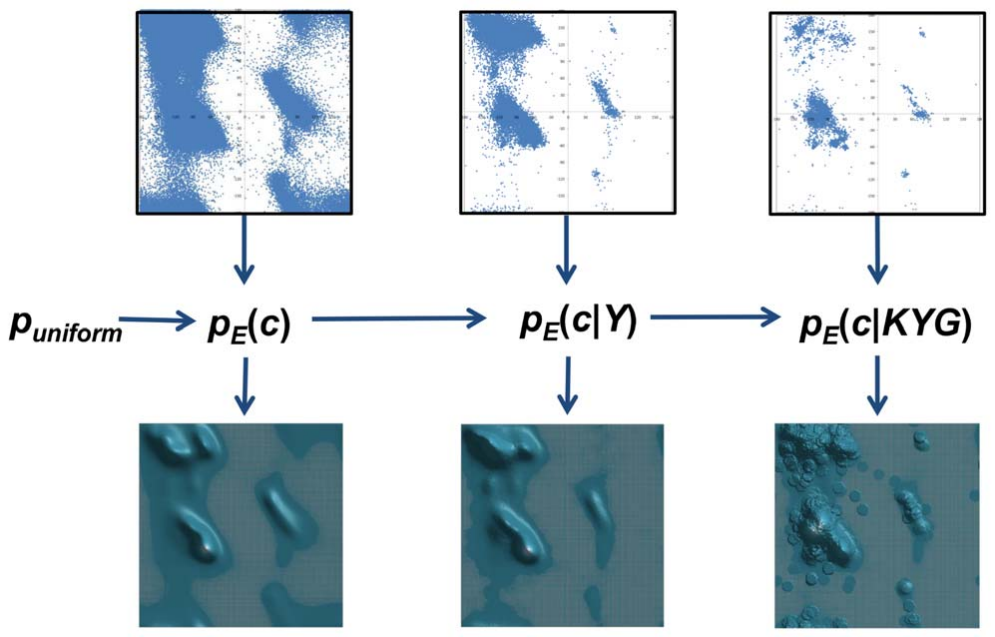
Illustration of the cascading probability estimation procedure. Raw phi-psi distributions (top row) are combined with prior probability distributions to form optimal PDFs (bottom row), with the goal of estimating *p_E_*(*c*|*KYG*), the phi-psi PDF of the central *Y* (tyrosine) residue flanked by *K* (lysine) and *G* (glycine). Initially, the sequence-independent PDF *p_E_*(*c*) was estimated by combining the raw distribution of the universe of structures with *p_uniform_*, the uniform distribution (i.e., state of maximum ignorance, 1/*b*, where *b* is the number of conformational states). The PDF *p_E_*(*c*) was then used in combination with raw data to estimate *p_E_*(*c*|*Y*), probability function for amino acid *Y*, which in turn was used in combination with raw data to estimate the triplet *p_E_*(*c*|*KYG*). The combinations are mediated by hybrid coefficients *γ*, as described in Eq.(7), which can be optimized automatically via the Information Maximization Device (IMD).

In the general case of formulating PDFs for the structure of the central residue of a triplet sequence *XYZ*, *p_E_*(*c*|*XYZ*), the raw count is buttressed by the structural PDF of the single amino acid *Y*, or *p_E_*(*c*|*Y*). The structural PDF of amino acid *Y*, *p_E_*(*c*|*Y*), can in turn be properly estimated by buttressing the raw counts for *Y* with the structural PDF describing the universe of structures (i.e., sequence-independent structural distributions), or *p_E_*(*c*). At this point, the universe of structures is arguably well-represented in the database, so that the raw frequency count at reasonable resolutions may be taken as an acceptable probability estimator. However, to ensure consistency and to guard against sparse data bias due to overpartitioning of the structural space, the PDF *p_E_*(*c*), is still estimated by buttressing raw counts with the uniform prior distribution, the state of maximum ignorance (i.e., all structural states are equally likely to occur). To summarize, for each level of sequence description, the equations describing the probability at a discrete structural state *c* are:
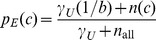
(7a)

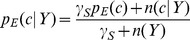
(7b)

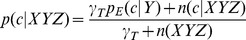
(7c)where 1/*b* is the uniform density function (*b* is the number of structural states in the phi-psi space), *γ_U_*, *γ_S_*, and *γ_T_* are hybrid coefficients, *n*(*c*) is the number of occurrences in the data set of conformation *c*, *n*(*c*|*s*) refers to the number of occurrences in the data set of sequence *s* in conformation *c*, and
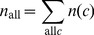
(7d)

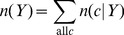
(7e)

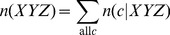
(7f)where the summation runs through all structural states. The data set {*c*,*s*}_E_ is used to generate these raw counts. These equations constitute a hierarchical procedure for estimating PDFs: the uniform distribution is used to estimate the structural PDF of the universe of structures *p*(*c*|*U*), which in turn is used to estimate the structural PDF for a single amino acid *p*(*c*|*Y*), which in turn is used to estimate the structural PDF for the central residue in a triplet sequence *p*(*c*|*XYZ*). See Figure 2 for an illustration of this hierarchic probability estimation procedure. It can be observed in Eq.(7a)–(7c) that as *n*_all_, *n*(*Y*), and *n*(XYZ) approach infinity (the point where virtually all natural protein sequences and structures are known), the *p* estimates reduce to the raw frequencies, as they ought to.

The probability equations contain three variables: *γ_U_*, *γ_S_*, and *γ_T_*. The optimal values for these variables, like any other adjustable parameter, can be derived computationally by using the IMD in Eq.(6). The principle followed is the same as for any other factor: the *γ* that maximizes mutual information is its optimal value. A numerical solution is achieved by a simple stepwise grid search for the maximum mutual information across a range of *γ* values.

### E. Data Sets and Specific Procedures

#### Data Sets

Four structural data sets were organized. The first set, called PDBSEL here, was taken from the PDB-SELECT list [20], composed of chains with a maximum of 25% pairwise sequence homology, downloaded from http://bioinfo.mni.th-mh.de/pdbselect/ in January 2012. Only chains with a resolution of 2.0Å or lower were included in PDBSEL, resulting in a data set composed of 3,205 chains, totaling 544,560 residues.

The second data set is a larger set of high-resolution structures. Called BLCLUST, it was organized from the entire Protein Data Bank (PDB) database, downloaded from http://www.rcsb.org/pdb/static.do?p=download/ftp/resources.jsp in January 2012. BLCLUST includes all chains in the PDB with resolution of 2.0Å or lower, and is partitioned by using the BLASTClust algorithm to make clusters of related sequences. Chains with at least 30% sequence similarity with equal to or greater than 90% alignment coverage are clustered together. BLCLUST contains a total of 77,838 protein chains, totaling 18,539,789 residues, grouped into 23,069 clusters.

The third data set is called BLC-NEW, composed of protein chains downloaded from the same resource as BLCLUST in November 2012. Chains already in BLCLUST were excluded. From each of the remaining clusters a single representative chain was identified and included in BLC-NEW. Thus, this data set is composed of newly solved structures of protein chains whose sequences are distinct from any found in BLCLUST, which was used in deriving the high-resolution phi-psi plots and the associated KBPs. Therefore, in this work, BLC-NEW serves as a testing set for an unbiased comparison between the methods described in this paper and other backbone torsion KBPs in literature. BLC-NEW is composed of 740 protein chains totaling 169,920 residues.

The fourth data set is called CASP10, composed of a diverse set of protein chains that were part of the 10^th^ Critical Assessment of Techniques for Protein Structure Prediction [31], a community-wide effort to independently assess structure prediction methods. The native structures of the 125 protein chains were gathered (with average length of 167 residues), along with an average of 367 decoys per protein chain, composed of high-resolution models submitted by various groups who participated in the assessment. The CASP10 data set, downloaded from http://predictioncenter.org/download_area/CASP10/, was used for extensive cross validation tests (in threading).

#### Partition of phi-psi space and frequency counts

**Figure 3:**
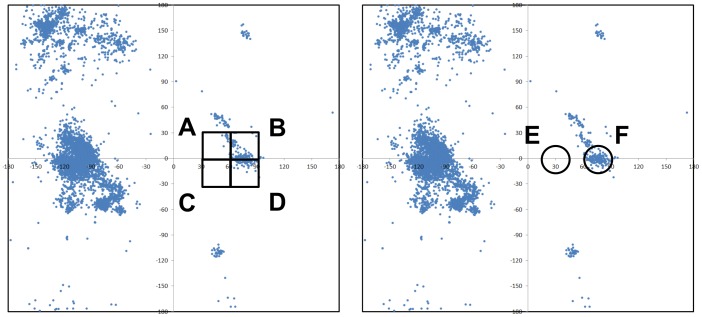
Partitioning the phi-psi space. The raw phi-psi plots of triplet *KYG* are shown. A small section in the plot on the left is partitioned by standard binning (at a resolution of 30 degree squares), while the same region of space on the right is partitioned by the dynamic radius approach, both weighted and unweighted (at a resolution of 30 degree diameter). The boundaries of standard binning are static, while the boundaries of dynamic radius depend on the location of the point being considered. For instance, in estimating the structural propensity around a point located in (75°,2°), the frequency value is that of square B for standard binning and circle F in dynamic radius. A slightly different data point at (75°,−2°) uses a much different frequency value of square D, while it will be nearly the same as circle F for dynamic radius (shifted 4 degrees towards the bottom). This example illustrates the significant frequency discontinuity between nearly similar locations when standard binning is used, compared to dynamic and weighted dynamic radius approaches. As another example, consider the data point (30°,2°). Standard binning assigns a non-negative frequency in square A due to the occurrences in the upper right corner of the bin. A much more accurate frequency estimate is made by circle E using dynamic radius, which reflects more realistic propensities around that given data point.

The phi-psi space was discretized in three ways: by standard binning, dynamic radius, and weighted dynamic radius (Figure 3). For standard binning, the 360°×360° space was subdivided into square bins of equal size depending on resolution, and frequency counts were obtained by counting the individual occurrences within each square bin, divided by the total number of data points. For dynamic radius, the frequency count at any point in the 360°×360° space was obtained by counting all occurrences within a specified radius *r*, divided by the total number of data points. For weighted dynamic radius, the contribution of each occurrence falling within a specified radius *r* to the frequency count at any point in the 360°×360° space was weighted inversely to its distance from the point, using the following cosine function:
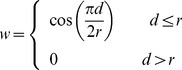
(8)where *d* is the distance from the point and *r* is the specified radius. This has the obvious effect of preferentially weighting data that occur very close to the point in question, resulting in smoother density functions.

#### Threading tests

Gapless threading was done to measure the effectiveness of KBPs in fold recognition. A large set of diverse sequences (short 10-mers as well as full-length chains from CASP10) were assembled. Each sequence was aligned with its correct (native) conformation as well as with a large set of decoy conformations. Each alignment of a given sequence *s* with the conformation *c* was scored with the triplet-sequence-dependent phi-psi angle-pair KBP:
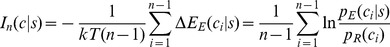
(9)where *n* is the sequence length, *s* is the triplet sequence surrounding the phi-psi angle pair *c_i_* in question, and *I_n_*(*c*|*s*) is the mean mutual information for the chain. The rank *r* of the score from the native conformation was computed by counting decoy scores larger than the native score. The mean percentile rank <*r*> was computed from repeated application of the threading exercise on all the sequences in the given data set (10-mers and CASP10).

Four sets of threading tests were done here. The purpose of the first two sets was to evaluate the fitness of the different KBPs arising from varying parameterization, probability estimation method, and training data set explored in this work. One threading test involved threading 5000 10-mer sequences and 5000×500 decoy conformations randomly picked from PDBSEL. The other threading test involved the CASP10 decoy set, composed of 125 chains (with average length of 175 residues). The purpose of the third and fourth threading sets was to compare the performance of the best KBPs derived here to those triplet energy functions derived by Betancourt [16]. The third threading test involved randomly picking out 5000 10-mer sequences from BLC-NEW and 5000×500 decoy conformations from PDBSEL, while the fourth threading test involved the same CASP10 decoy set.

## Results and Discussion

The iterative structure of the Information Maximization Device (IMD), this work's operative optimization scheme, is expressed in Figure 1. The optimization requires two representative data sets—the testing and the training data sets—as well as a procedure to estimate probability empirically from (training) data, together with a way to define structural resolution that maximizes information. Results are discussed below.

### A. Probability Estimation

Probability estimation from empirical data was achieved by a hierarchic estimation method, as described in Section D of the Materials and Methods section. In this work, the sequence-independent PDFs (i.e., the structural distribution of the universe of amino acids in folded proteins) was formed first, followed by 20 amino acid-specific PDFs, and then finally 8000 triplet PDFs. The hybrid coefficient *γ* modulates the effect of the distributions being combined—small values favor the raw distributions while large values favor the prior distribution; its optimum value is that which produces PDFs that contain maximal information. A stepwise search across a wide range of *γ* accomplishes this simple optimization.

**Figure 4:**
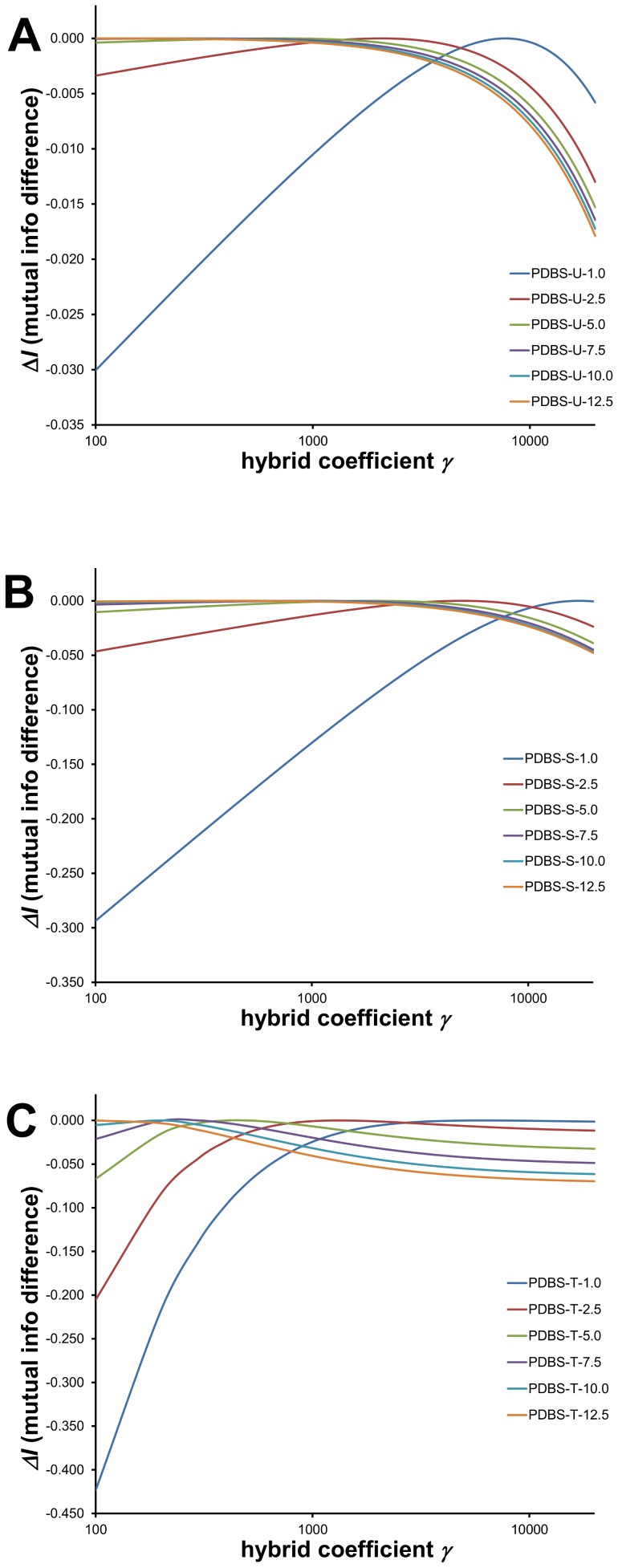
Optimization of hybrid coefficient *γ*. We illustrate the effect of the hybrid coefficient on mutual information using PDBSEL data set and dynamic radius space partitioning approach. A range of values of *γ*, on the x-axis, were used to formulate probability estimates using Eq.(7). The y-axis is the difference in mutual information from the maximum value for every resolution, i.e. maximum mutual information occurs at Δ*I* = 0. For each set of plots, different resolutions are shown (identified by the number in the legends). Optimization of three hybrid coefficients are shown: (A) *γ*_U_ for estimating sequence-independent PDFs (Eq.(7a)); (B) *γ*_S_ for estimating single amino acid PDFs (Eq.(7b)); and (C) *γ*_T_ for estimating triplet PDFs (Eq.(7c)). The parabolas make it straightforward to identify optimal *γ* values that yield maximum mutual information.

The graphs in Figure 4 illustrate the variation in *I_E_*(*C*,*S*) across different values of *γ*. In these graphs, another variable being explored is the resolution of the PDFs, discussed in a later section. But it's worth noting now that since resolution is critical in the formulation of the PDFs, mutual information depends significantly on resolution, as shown in these graphs. PDFs of higher resolution require more raw data, so that the role of the prior distribution becomes increasingly prominent. For each set of conditions, the value of the hybrid coefficient follows a simple parabola, making it straightforward to identify the maximum. The wide variation in optimal *γ* values demonstrates that using an arbitrarily set hybrid coefficient without regard to data conditions, such as Sippl's information quantum [22], may not lead to the best PDFs or knowledge-based potentials (KBPs). The need to optimize *γ* becomes especially acute at the extremes—when data are abundant (i.e., at lower resolutions) or when data are scarce (at high resolutions).

### B. Effect of phi-psi Space Partition, Resolution, and Database Size on Mutual Information Estimate

The effect of three factors on the quality of single sequence and triplet sequence phi-psi plots was examined.

First, three different approaches to partitioning the phi-psi space were applied—standard binning, dynamic radius, and weighted dynamic radius (Figure 3). Standard binning is the most common and also the simplest way to discretize structural space, but artificially drawn boundaries can potentially create jagged and unphysical distributions. In contrast, the dynamic radius approach works to smooth out such sharp disjunctions: the frequency at any point across the phi-psi space is computed by counting all occurrences within the specified radius. Weighted dynamic radius works the same way, except that each occurrence is weighted inversely to its distance from the point in question using the cosine function (Eq.(8)).

Second, within each partition, a range of resolutions was explored: for standard binning, the side length of square bins ranged from 2 degrees to 60 degrees; for dynamic radius, the radius of the circle ranged from 1 degree to 20 degrees; and for weighted dynamic radius, the radius ranged from 2.5 degrees to 60 degrees.

Third, two training data sets were used for {*c*,*s*}_E_ to derive the probability distributions, PDBSEL and BLCLUST, described in detail in Section E of the Materials and Methods section. The testing data set {*c*,*s*}_T_ employed for the evaluation of *I_E_*(*C*,*S*) was always PDBSEL. Comparing {*c*,*s*}_T_ and {*c*,*s*}_E_, the composition of PDBSEL overlaps completely with itself, and also significantly overlaps with BLCLUST. Thus, in order to avoid bias (due to complete memorization), a jackknife method was applied, as described in Section D of the Materials and Methods section. Briefly, in order to preserve the integrity of the Information Maximization Device (Eq.(6) and Figure 1), each term in the summation, signifying one chain in BLCLUST, is evaluated by removing that chain from the training set {*c*,*s*}_E_, be it PDBSEL or BLCLUST. Again, it is worth repeating that this work's information-theoretic analysis reveals that {*c*,*s*}_E_ and {*c*,*s*}_T_ need not be identical or even related data sets. The only caveat is to ensure that no overlap exists in the two data sets so that an unbiased measurement of mutual information of PDFs and performance of KBPs can be made.

Because the data set BLCLUST, a comprehensive set of all high-resolution chains in the PDB, contains many similar and near-identical sequences and conformations, a computational adjustment has to be made. The contribution of each chain in a cluster of similar/near-identical sequences was weighted accordingly: 
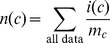


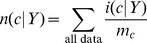
(10)

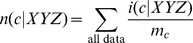
where *i* = 1 if the data point has structure *c* and the specified sequence (whether no sequence, amino acid *Y*, or triplet *XYZ* respectively), or *i* = 0 otherwise; and *m_c_* is the number of chains in the cluster in which the data point belongs. This ensures that clusters with many chains do not dominate the resulting raw distributions. The advantage of this approach, compared to picking out only one representative per cluster, is that it considers the variation within the cluster and incorporates whatever information might be contained into more refined PDFs. For instance, structure variation observed in chains of identical sequences (e.g. identical subunits of multimeric proteins) will be considered as a demonstration of structural propensity, not discarded as “noise.” Finally, it should be mentioned that in the jackknife procedure as applied to BLCLUST, *all* chains in the homologous cluster are eliminated from each term in the summation in Eq.(9).

**Figure 5:**
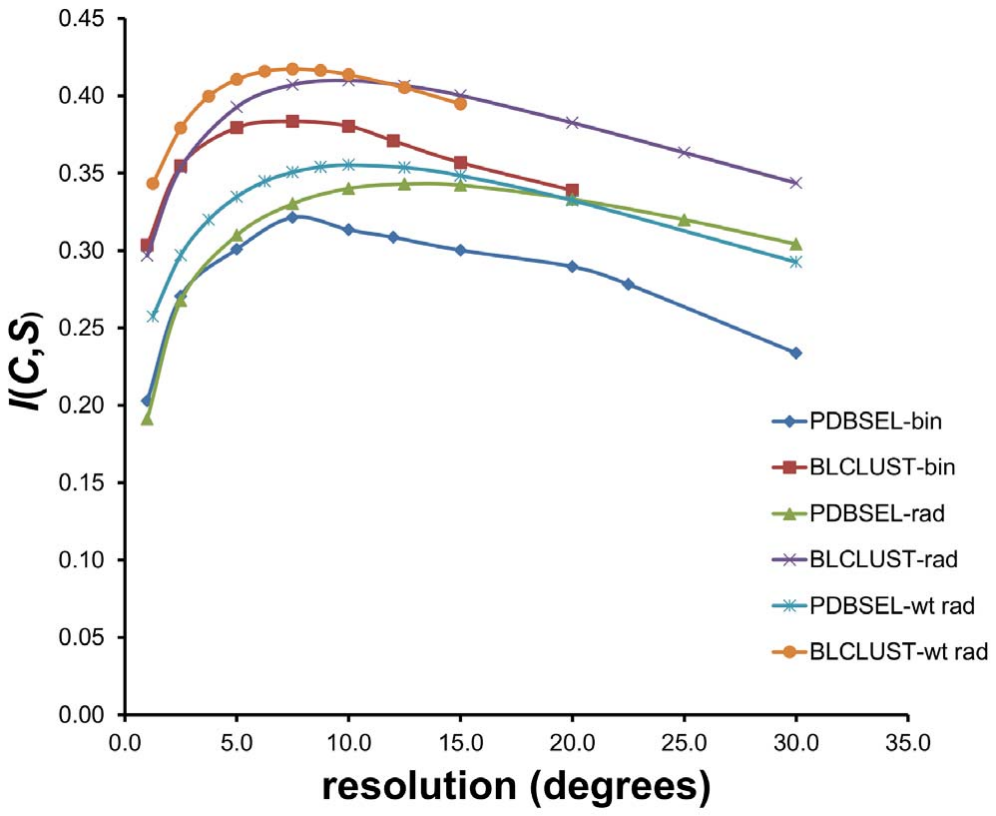
Mutual information values of PDFs derived by IMD for different sets of conditions. The calculations involve two training data sets (PDBSEL and BLCLUST), three partitioning approaches (bin  =  standard binning; rad  =  dynamic radius; wt rad  =  weighted dynamic radius), and a range of resolutions (from 2.5° to 30.0°). It is easy to identify the conditions that yield the maximum mutual information overall: it is the set of PDFs derived from BLCLUST using weighted dynamic radius at a resolution of 15° (radius).

Results of the optimization of the single amino acid and triplet sequence PDFs under different states for these three factors—partition of the phi-psi space, resolution, and database size—are given in Table 1. Plotting *I_E_*(*C*,*S*) for triplet sequences across the range of resolutions for three different partitions and two databases in Figure 5 allows the following observations. First, an optimal resolution for each set of factors exists—between a state of excessively high resolution that cannot be supported by the size of current data and a state of low resolution that washes away specific structural information in sequence. These optimal resolutions vary depending on the combination of other factors—i.e., 15 degree squares for standard binning, circles with radius of 10–12.5 for dynamic radius, and circles with radius of 15–20 for weighted dynamic radius. The ranges of *I_E_*(*C*,*S*) across the span of resolutions examined are wide—from a low of 0.19 nats to a high of 0.42 nats.

**Table 1:** Results of IMD for different space partitions, resolution, and database size.

	bin size (degrees)	*I_E_*(*C*,*Y*) (singlet *I*, nats)	*I_E_*(*C*,*XYZ*) (triplet *I*, nats)	*I_E_*(*C*,*X_Z*) (flanking *I*, nats)	*γ*_U_ (Eq.7a)	*γ*_S_ (Eq.7b)	*γ*_T_ (Eq.7c)
**PDBSEL, standard binning**	2.0	0.199	0.203	0.004	6530	14726	4975
	5.0	0.255	0.270	0.016	1808	4396	1141
	10.0	0.266	0.301	0.035	639	1880	419
	15.0	**0.273**	**0.321**	0.049	369	1173	249
	20.0	0.257	0.313	0.056	247	814	180
	24.0	0.247	0.309	0.062	160	645	147
	30.0	0.235	0.300	0.066	132	524	118
	40.0	0.223	0.290	**0.067**	84	349	92
	45.0	0.216	0.278	0.063	65	318	83
	60.0	0.177	0.234	0.057	38	218	73
**PDBSEL, dynamic radius**	1.0	0.188	0.191	0.003	7720	17254	6123
	2.5	0.254	0.268	0.014	2066	4943	1324
	5.0	0.275	0.310	0.035	701	1908	450
	7.5	**0.279**	0.330	0.052	280	984	251
	10.0	0.276	0.340	0.064	135	608	167
	12.5	0.270	**0.343**	0.073	72	406	123
	15.0	0.264	0.342	0.078	23	277	97
	20.0	0.250	0.333	**0.083**	4	153	67
	25.0	0.237	0.320	**0.083**	3	84	51
	30.0	0.223	0.304	0.081	1	57	41
**PDBSEL, weighted dynamic radius**	2.5	0.248	0.257	0.009	1117	6434	2054
	5.0	0.272	0.297	0.025	198	2504	677
	7.5	0.279	0.320	0.040	56	1324	368
	10.0	**0.281**	0.335	0.053	27	805	242
	12.5	**0.281**	0.345	0.064	14	526	175
	15.0	0.279	0.351	0.072	4	352	136
	17.5	0.276	0.354	0.078	1	250	109
	20.0	0.272	**0.355**	0.083	1	183	91
	25.0	0.264	0.354	0.089	1	101	67
	30.0	0.256	0.348	0.092	1	66	53
	40.0	0.239	0.332	**0.093**	1	17	36
	60.0	0.207	0.293	0.086	1	3	22
**BLCLUST, standard binning**	2.0	0.279	0.303	0.024	670	6229	2842
	5.0	**0.292**	0.355	0.062	45	1067	808
	10.0	0.287	0.379	0.092	1	260	352
	15.0	0.279	**0.384**	0.104	1	99	223
	20.0	0.272	0.380	0.109	1	44	162
	24.0	0.260	0.371	**0.111**	1	15	135
	30.0	0.247	0.357	0.110	1	12	108
	40.0	0.234	0.339	0.105	1	11	85
**BLCLUST, dynamic radius**	1.0	0.276	0.297	0.021	1008	7781	3365
	2.5	0.295	0.354	0.059	42	1293	915
	5.0	**0.297**	0.393	0.095	1	285	367
	7.5	0.294	0.407	0.113	1	89	218
	10.0	0.289	**0.410**	0.121	1	37	150
	12.5	0.282	0.407	**0.125**	1	19	111
	15.0	0.275	0.400	**0.125**	1	7	87
	20.0	0.261	0.383	0.122	1	1	59
	25.0	0.247	0.363	0.116	1	1	43
	30.0	0.234	0.344	0.110	1	1	34
**BLCLUST, weighted dynamic radius**	2.5	**0.301**	0.343	0.042	12	1666	1391
	5.0	0.300	0.379	0.079	10	402	539
	7.5	0.299	0.400	0.101	1	136	317
	10.0	0.297	0.411	0.114	1	56	218
	12.5	0.294	0.416	0.122	1	25	162
	15.0	0.290	**0.417**	0.127	1	14	126
	17.5	0.286	0.416	0.130	1	6	101
	20.0	0.282	0.414	**0.132**	1	1	83
	25.0	0.274	0.405	**0.132**	1	1	60
	30.0	0.265	0.395	0.130	1	1	45

Second, the weighted dynamic radius partitioning is slightly superior to dynamic radius partitioning, while both are substantially superior to standard binning. The maximum *I_E_*(*C*,*S*) for standard binning reaches only 0.32 nats for PDBSEL and 0.38 nats for BLCLUST, while the dynamic radius reaches 0.34 and 0.41 nats respectively, and weighted dynamic radius reaches 0.36 and 0.42 nats respectively.

Third, PDFs constructed from BLCLUST carry considerably more mutual information than those from PDBSEL. The maximum *I_E_*(*C*,*S*) extracted from BLCLUST is 0.42 nats, which is 17% higher than the maximum *I_E_*(*C*,*S*) from PDBSEL, at 0.36 nats.

A dissection of the triplet *I_E_*(*C*,*S*) yields several observations. Table 1 lists mutual information values resulting from both single amino acid *I_E_*(*C*,*Y*) and triplet sequence *I_E_*(*C*,*XYZ*). The additive nature of mutual information [30] suggests a way to isolate the specific effect of the flanking residues on the phi-psi conformation of the central residue, *I_E_*(*C*,*X_Z*), as follows:
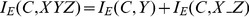
(11)


The informatic benefit of including the flanking residues in defining backbone conformation can be measured simply by the increase in the amount of mutual information as one moves from single amino acid to triplet sequence description. In all conditions examined (Table 1), *I_E_*(*C*,*Y*) is always higher than *I_E_*(*C*,*X_Z*), meaning that the effect of the central residue on its backbone conformation is greater than the effect of its flanking residues.

Maximum values of both *I_E_*(*C*,*Y*) and *I_E_*(*C*,*X_Z*) for each set of data in Table 1 are highlighted in bold. Values for *I_E_*(*C*,*Y*) range from 0.273 to 0.301 nats, which is narrower than the that exhibited by *I_E_*(*C*,*X_Z*), which ranges from 0.067 to 0.125 nats. It appears that at the current volume of data, phi-psi PDFs for single amino acids are less sensitive to the factors examined, implying that differing approaches to formulating these PDFs would yield roughly the same amount of mutual information. Triplet PDFs, on the other hand, appear to be acutely dependent on the size of the data set. This is consistent with the fact that triplet PDFs require a larger amount of data. A better mapping of the effect of the flanking residue *I_E_*(*C*,*X_Z*) is the primary advantage of this information-based approach.

Looking at the effect of resolution on mutual information yields a similar observation. Maximum values for *I_E_*(*C*,*Y*) occur at a much higher resolution than the maximum values for *I_E_*(*C*,*X_Z*), indicating that the volume of current data is sufficient to elucidate low-probability regions of the phi-psi space in single amino acids but not for triplets. It follows that as new structures are deposited into the PDB, mutual information of triplet PDFs will increase significantly along with resolution.

**Figure 6:**
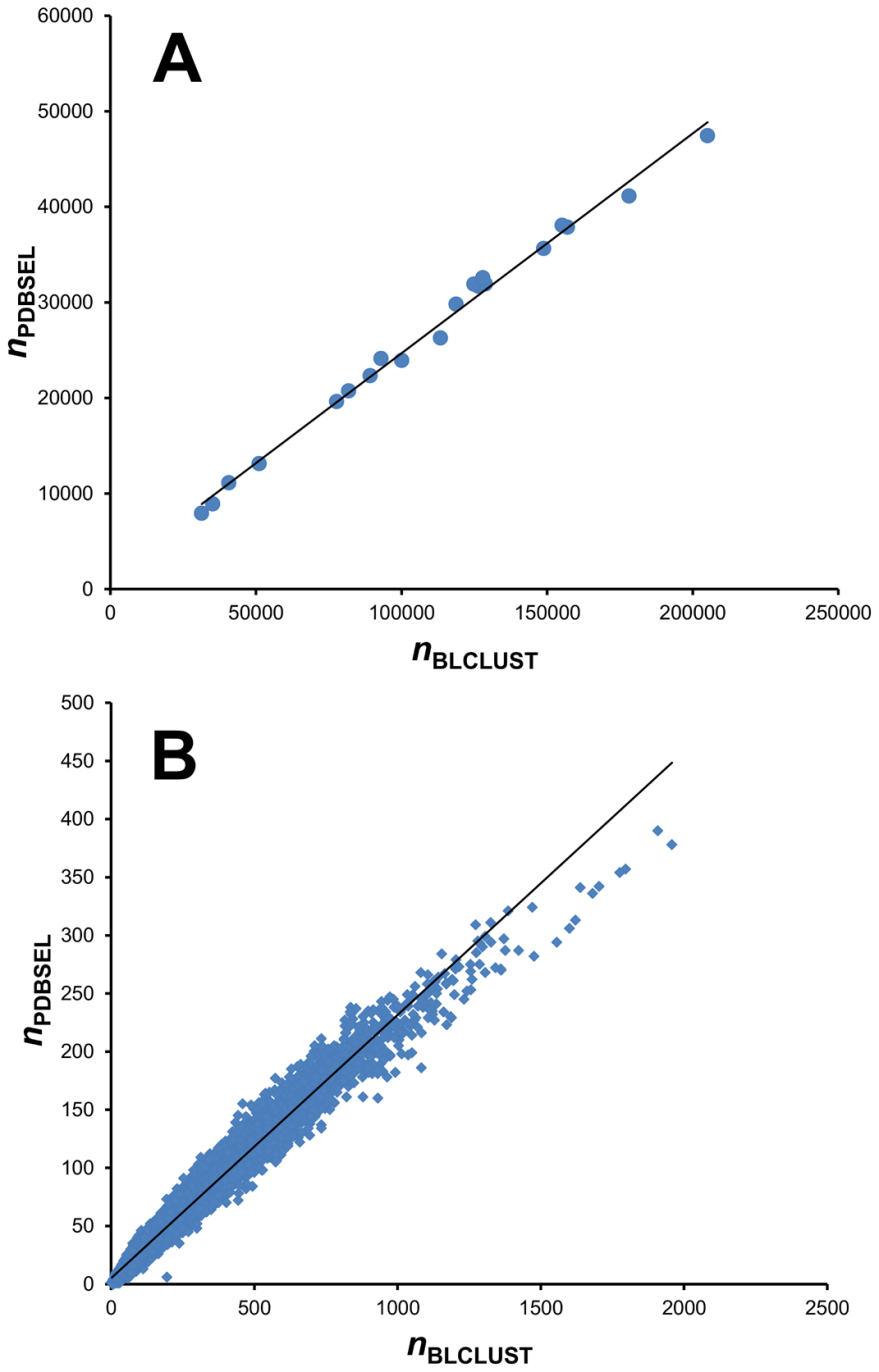
Number of occurrences of each of the 20 amino acids and 8000 trimers in two training data sets used, PDBSEL and BLCLUST. These plots were made to investigate the nature of the increase in sequence representation as one goes from PDBSEL to BLCLUST. The variable *n*_PDBSEL_ is the number of instances found in PDBSEL, while the variable *n*_BLCLUST_ is the number of equivalent data points found in BLCLUST. (Equivalent data points is the count of all the occurrences in BLCLUST weighted by *m_i_*, as described in Eq.(10).) (A) There are 20 points in the plot representing 20 amino acids. (B) There are 8000 point in the plot representing 8000 triplet sequences. Both plots show a strong linear relationship, showing that the relative proportions of 20 amino acids and 8000 triplet sequences are virtually the same in PDBSEL as in BLCLUST, and the increase in equivalent data points in BLCLUST is proportionally distributed across all amino acids and triplet sequences.

To explore the nature of the significant increase in mutual information from PDBSEL to BLCLUST, the numbers of observations for each data set were plotted for all 20 amino acids (Figure 6A). The strongly linear plot (*R* ^2^ = 0.99) shows that the increase in the number of data points for each of the amino acids due to an expanded data set scales with the original non-redundant population, which indicates that the relative amino acid distributions are roughly the same. Figure 6B, which plots the populations for all 8000 triplets, shows the same linear pattern (*R* ^2^ = 0.97). These two graphs show that the relative distributions of single amino acids and triplets are virtually the same in PDBSEL and BLCLUST, and that using BLCLUST increases the population of all single amino acids and triplets generally by more than four times. The resulting improvement in mutual information occurs not because PDBSEL is missing significant representation for some amino acids or triplets, but because we achieve more data representation in BLCLUST across the board.

### C. Comprehensive threading results

To test their utility, the optimally generated PDFs were incorporated into KBPs (via Eq.9), which were then applied to comprehensive fold recognition tests via threading. Two threading tests were implemented: a local threading procedure using short sequences and a full chain threading using the diverse CASP10 decoy set. These two tests involved the alignment of sequences onto structures from a large set of decoys, and then scoring the alignments with the respective KBP. The score of the alignment of the sequence onto its native conformation was then ranked among the decoy scores. A mean percentile rank <*r*> was computed from repeated threading of all sequences in the respective data sets.

**Figure 7:**
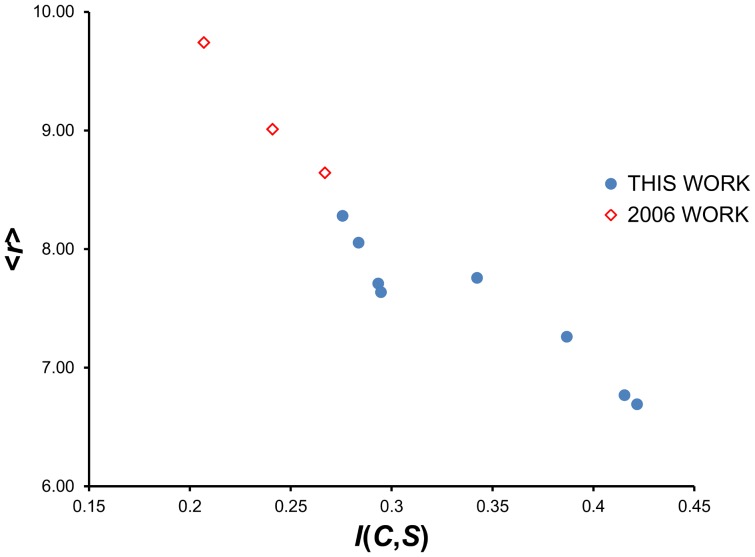
The linear relationship between mutual information *I*(*C*,*S*) and performance in fold recognition of 10-mer segments as measured by the mean percentile rank <*r*>. This plot illustrates the utility of the IMD in increasing performance of KBPs that use optimal PDFs in fold recognition (threading) on short 10-mer segments. (See Table 2 for a summary of the results for 10-mer threading.) Each of the circular points in the plot represents a set of PDFs optimized in this work under a distinct set of factors (training data set used, resolution, space partition). The diamond points are from an earlier work [23], included here to demonstrate that mutual information maximization can span different conditions and factors to be optimized yet still show strict linear relationship with performance. The strong correlation (seen here and in Figure 8) demonstrates that increasing mutual information estimates, by the direct manipulation of factors, is a viable strategy for creating more accurate PDFs and formulating KBPs that show improved fold recognition.

**Figure 8:**
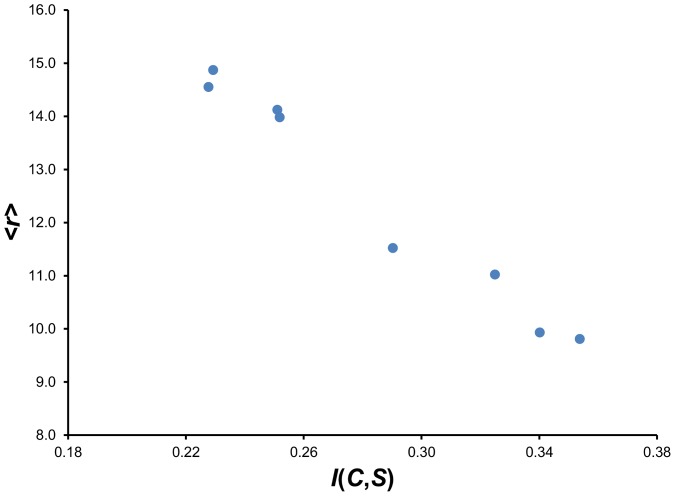
The linear relationship between mutual information *I*(*C*,*S*) and performance in fold recognition of CASP10 chains as measured by the mean percentile rank <*r*>. This plot illustrates the utility of the IMD in increasing performance of KBPs that use optimal PDFs in fold recognition (threading) on 125 CASP10 chains. (See Table 2 for a summary of the results for CASP10 threading.) Each of the points in the plot represents a set of PDFs optimized in this work under a distinct set of factors (training data set used, resolution, space partition). The strong correlation (seen here and in Figure 7) demonstrates that increasing mutual information estimates, by the direct manipulation of factors, is a viable strategy for creating more accurate PDFs and formulating KBPs that show improved fold recognition.

Figure 7 (local threading) and Figure 8 (CASP10 decoy threading), along with Table 2, show the results of comprehensive threading for selected KBP conditions. The mean percentile rank <*r*> for each KBP is plotted against the mutual information of the PDF used to construct the KBP. A robust linear correlation can be observed in both plots, consistent with previous findings [23], [24] and reinforces the principle that mutual information maximization is an effective way to optimize KBPs. The two best performing KBPs that were built from PDFs assembled from BLCLUST—the weighted dynamic radius approach with resolution at 15 degrees (radius), and the dynamic radius approach with resolution at 10 degrees (radius)—are also the two that carry the highest *I_E_*(*C*,*S*) among all PDFs examined, proving that maximizing *I_E_*(*C*,*S*) is an effective strategy for optimizing KBP performance in fold recognition.

**Table 2:** Threading results for various KBPs using PDFs of different conditions.

		PDBSEL-radius^a^		BLCLUST-bin^b^		BLCLUST-radius^c^		BLCLUST-wt rad^d^	
		single	triplet	single	triplet	single	triplet	single	triplet
**10-mer threading test**	*n* 10-mer^e^	1265	1265	1265	1265	1265	1265	1265	1265
	n decoys^f^	5000	5000	5000	5000	5000	5000	5000	5000
	<*r*>^g^	8.28	7.76	8.05	7.26	7.71	6.77	7.63	6.69
	<*I*-nat>^h^	0.276	0.342	0.284	0.387	0.293	0.416	0.295	0.422
	<*I*-dec>^i^	−0.305	−0.411	−0.334	−0.483	−0.328	−0.501	−0.328	−0.508
	*J* j	0.581	0.753	0.617	0.870	0.622	0.916	0.623	0.929
**CASP10 threading test**	n chains^k^	125	125	125	125	125	125	125	125
	n decoys^l^	367	367	367	367	367	367	367	367
	<*r*>^m^	14.87	11.52	14.55	11.02	14.12	9.93	13.98	9.81
	<*I*-nat>^h^	0.229	0.290	0.228	0.325	0.251	0.340	0.252	0.354
	<*I*-dec>^i^	0.060	0.029	0.053	0.029	0.072	0.008	0.072	0.013
	*J* j	0.169	0.261	0.175	0.296	0.179	0.332	0.179	0.341

a KBP formed with PDBSEL training data set and dynamic radius space partition.

b KBP with BLCLUST and standard binning.

c KBP with BLCLUST and dynamic radius.

d KBP with BLCLUST and weighted dynamic radius.

e The number of 10-mer segments picked randomly from the data set and subjected to threading test.

f The number of random decoys per chain.

g The percentile rank of the native conformation score amidst 5000 decoy conformation scores.

h The mean mutual information score (Eq.9) of the native conformation.

i The mean mutual information score computed for decoy conformations.

j The total divergence score, an information-theoretic quantity defined as <*I*-nat> - <*I*-dec>, which measures the mean gap between native scores and incorrect scores (see Ref. 23).

k The number of chains in the CASP10 set. The average chain length is 175 residues.

l The average number of decoys per chain in the CASP10 set.

m The percentile rank of the native conformation score amidst the decoy conformation scores in the CASP10 set.

### D. Nature of Optimized phi-psi Plots

**Figure 9:**
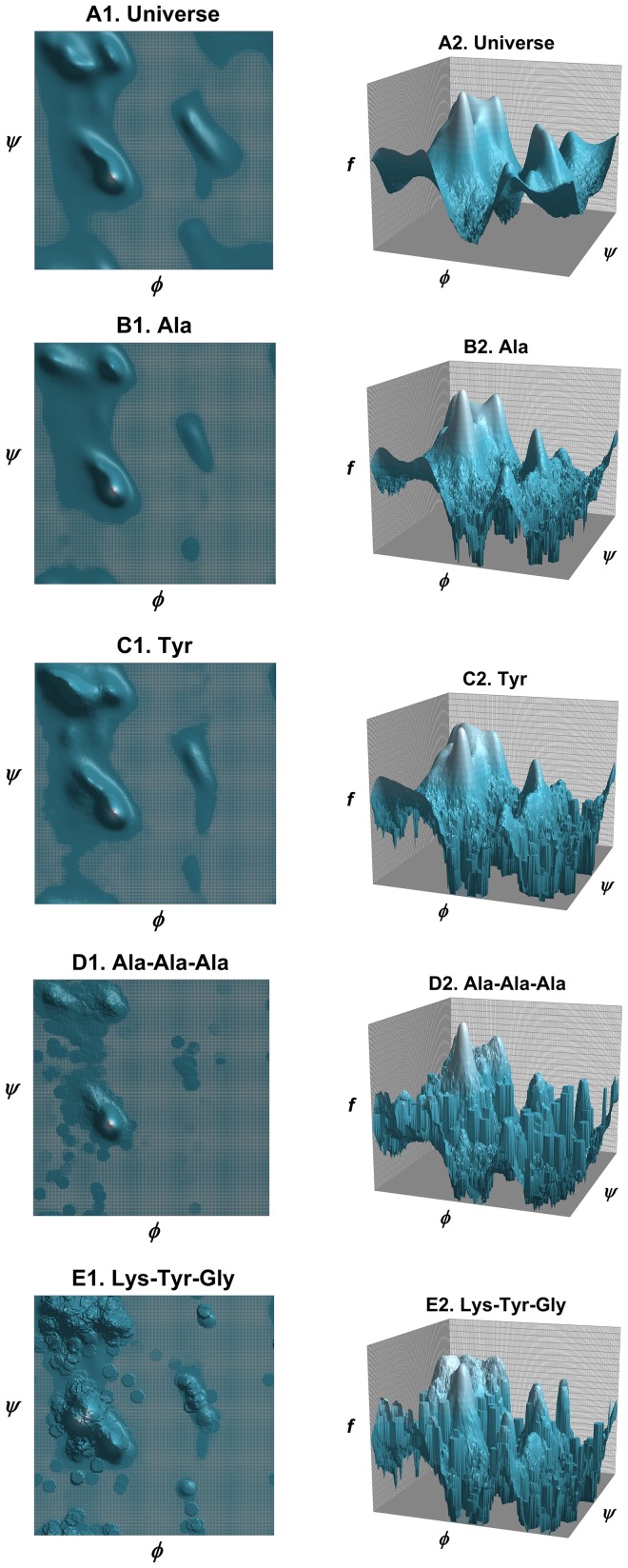
Optimal Ramachandran phi-psi plots. Some examples of the phi-psi plots generated by the IMD approach: (A) the universe of structures (sequence-independent distribution); (B) Alanine; (C) Tyrosine; (D) Alanine-Alanine-Alanine tripeptide; and (E) Lysine-Tyrosine-Glycine tripeptide. Each of these distributions is illustrated in two ways: with standard 2D plot and with a 3D log plot. The *z*-axis of the log plot is the frequency *f*. (The frequency range of the topmost level on the *z*-axis is 0.5–1.0, the penultimate level is 0.25–0.5, the third level is 0.125–0.25, and so on.) Smooth contours in log plots show well-represented and well-defined regions, while grainy contours mark regions that require more data.

The probability at any point in phi-psi space can be computed in a straightforward way using the dynamic radius and weighted dynamic radius approaches. Those displayed in Figure 9 are typical plots that result from these approaches using BLCLUST data.

Regions that give rise to regularity in secondary structure (α, β, α_L_) are typically the most represented in these plots. The details in fringes and the outlying regions—the low probability conformations—are what is lacking in resolution. These can be observed in the plots in Figure 9, where 3D log plots have been used to exaggerate the coarseness of low probability regions. (The *z*-axis, representing propensity *p*, is in log scale.) Smooth contours in log plots show well-represented and therefore well-defined regions, while grainy contours mark regions that require more data.

The sequence-independent phi-psi plot is largely smooth and well-defined. The amino acid-specific plots show more grainy areas outside regions that form regular secondary structure. The plot for Ala (Figure 9B), formed by 178,147 equivalent data points, is noticeably smoother than the plot for Tyr (Figure 9C), formed by only 77,718 equivalent data points. (Equivalent data points is the count of all the occurrences in BLCLUST weighted by *m_i_*, as described in Eq.10.) It should be noted that even with an abundance of equivalent data points, Ala appears to still be in need of resolution especially in low probability regions. Updating these plots as the PDB continues to grow will provide a more accurate picture of structural propensity across all regions of the phi-psi space.

As expected, coarser plots are formed for the triplet sequences as shown by representative examples in Figure 9. The triplet Ala-Ala-Ala (Figure 9D) has 1909 equivalent data points, among the most represented triplets in BLCLUST, and shows a clear propensity for alpha helical conformations, with a probability value of 0.55 at the highest point in the plot. As another example, the triplet Lys-Tyr-Gly (Figure 9E) does not appear to show the same structural propensities, and with only 407 equivalent data points, is especially coarse. The peak of the plot also occurs at the alpha helical region but at significantly lower probability (at 0.10).

Another significant advantage of using an expanded data set such as BLCLUST over PDBSEL is the increased representation in particularly rare triplets. In the PDBSEL data set, four triplets do not occur at all: Trp-Met-Trp, Cys-Trp-His, Met-Trp-Cys, and Trp-Trp-Trp. These occur in BLCLUST, and statistics show that these four triplets are represented by 8.35, 8.85, 4.50, and 5.32 equivalent data points respectively. Meanwhile, all triplets in BLCLUST are represented, with the rarest triplet in BLCLUST being Cys-Met-Trp, with 2.01 equivalent data points.

A point must be made to qualify the PDFs derived in this work. In an effort to use as much structural data as is available in the PDB, this initial study uses the over-all crystallographic resolution as the main criterion to select proteins for inclusion in the PDBSEL and BLCLUST data sets. However, accuracy of the atomic coordinates varies within the same structure (as indicated by the B-factor), so that the resulting phi-psi maps are actually aggregates of conformations of varying quality. Moreover, other factors exist that can potentially alter the backbone structure propensities expressed in the PDFs. These include intermolecular crystal packing interactions that affect exposed regions and variable loops of the protein molecule [32], missing atoms and residues in the model, as well as the existence of small molecules and ligands that are included in the crystal structure. The implicit assumption taken by comprehensive structural surveys such as this work is that specific deformations and deviations do not occur systematically, and therefore average out in a mean-force analysis [22]. However, a closer examination of the effects of these data conditions should be undertaken to explore how well the phi-psi maps produced here signify actual propensities of triplets (in the context of the folded protein) as they operate in nature. Due to its flexibility in exploring the effect of any structurally relevant variable or factor, the Information Maximization Device may also be employed in deriving optimal PDFs of coherent subsets of data (partitioned with respect to the factors identified above). It is critical that more stringent data collection criteria be balanced with the potential diminishment of the extracted mutual information, a situation where the application of IMD is well-suited. These issues need to be explored in future work.

### E. Comparing BLCLUST with BETAN in Fold Recognition

To test the utility of the PDFs proposed in this work, a direct comparison was conducted between Betancourt's local triplet backbone KBP [16] (called BETAN here) and the KBP derived from optimal PDFs built from using the comprehensive BLCLUST data set. For a fair comparison, an entirely new testing data set of high-resolution protein chains was assembled to be independent of the data sets used to derive both BLCLUST and BETAN potentials. This new data set consists of the most recent entries in the PDB that do not share any significant sequence similarities with chains belonging to either BLCLUST or BETAN. This data set, composed of 740 chains totaling 169,920 residues, is referred to as BLC-NEW.

**Figure 10:**
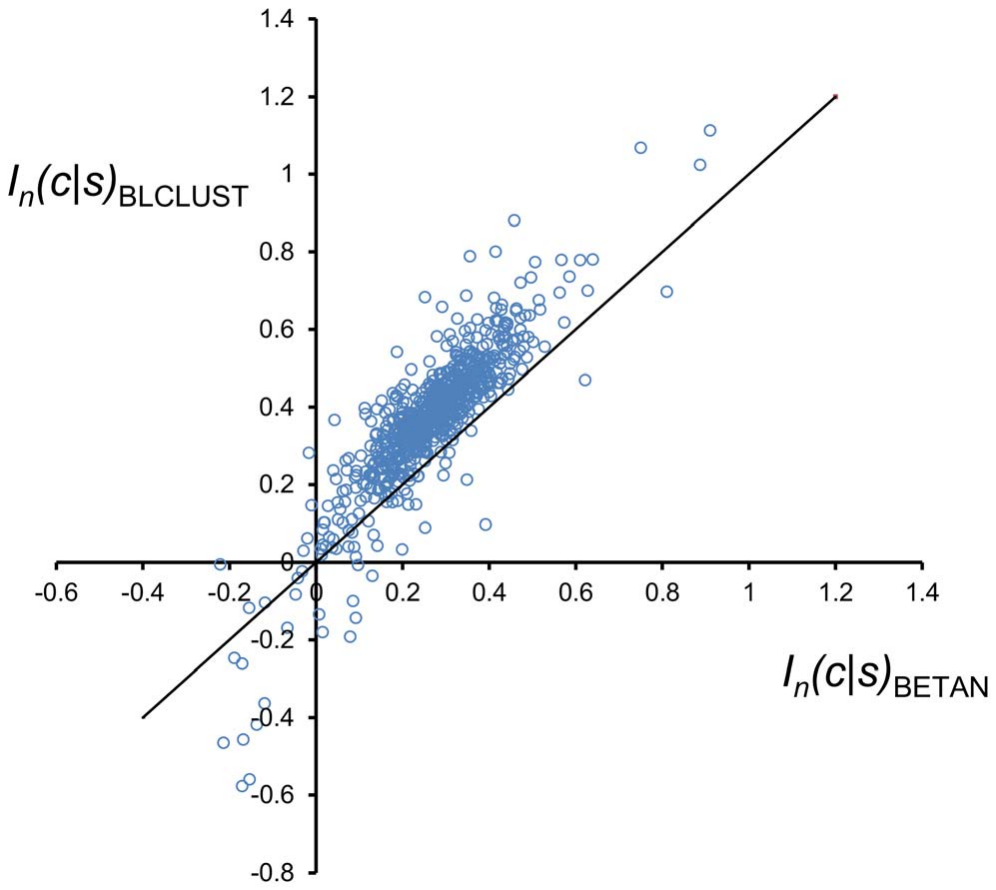
Comparison of mutual information scores for whole protein chains assigned by triplet BETAN and BLCLUST KBPs. Two scores were computed for the native structures of all 740 proteins chains in BLC-NEW using the score function *I_n_*(*c*|*s*), as defined by Eq.(9). One score is computed from optimal triplet PDFs derived from BLCLUST (using weighted dynamic radius, at resolution 15.0°), and another score is computed from BETAN PDFs. These two scores are plotted here. More than 93% of the protein chains appear above the diagonal line, which means their native conformations are scored higher by BLCLUST than by BETAN score functions. The assignment of high scores to native conformations is one desirable characteristic of a good score function.

The mutual information score was computed for the native conformation of each chain in BLC-NEW using KBPs derived from both BLCLUST and BETAN using Eq.(9) (with *n*  =  length of the protein chain). The results are plotted in Figure 10. Points that lie above the diagonal line are chains that have been assigned higher scores by BLCLUST compared to BETAN. Results show that the native conformations of nearly all chains (93.65%) exhibit higher mutual information scores in BLCLUST than in BETAN. Recalling the direct relationship between mutual information of PDFs and performance of their associated KBPs in fold recognition, these strong numbers point to the clear superiority of the PDFs derived in this work. In particular, the ability of a score function to assign high scores to native conformations points to the ability by the KBP function to detect nativeness.

Another way to examine the effectiveness of BLCLUST-derived PDFs is to measure the amount of mutual information that is caused specifically by the flanking residues of the triplet sequence. Analogous to Eq.(11), one can think of the *XYZ* triplet mutual information score as a combination of the contribution of the central amino acid *Y* and the flanking residue *X_Z* to the conformation *c*:
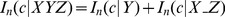
(12a)


The components of the score function can be derived by a simple expansion:
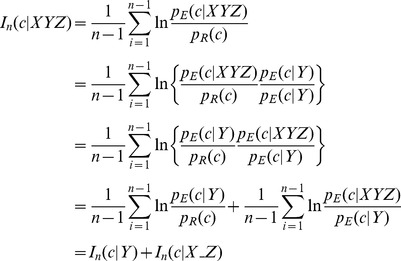
(12b)


This equation decomposes the contribution of the central residue and the flanking residues of the triplet. The goal here is to estimate the specific effect of including the flanking residues in the local KBP.

**Figure 11:**
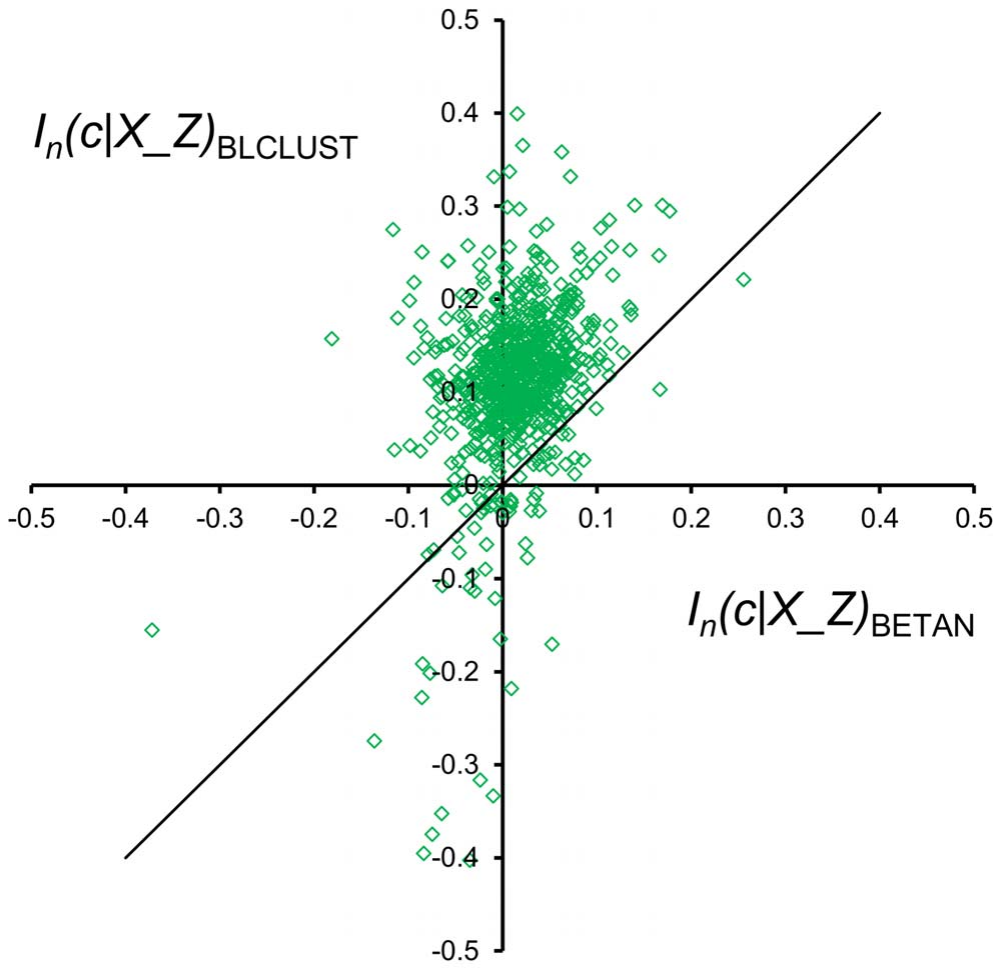
Comparison of mutual information scores for whole protein chains brought about by the flanking residues as assigned by triplet BETAN and BLCLUST KBPs. Eq.(12) is used to compute *I_n_*(*c*|*X_Z*), the portion of the triplet score that can be attributed to the influence of flanking residues on the phi-psi conformation of the central residue. One score is computed from optimal triplet PDFs derived from BLCLUST (using weighted dynamic radius, at resolution 15.0°), and another score is computed from BETAN PDFs. These two scores are plotted here. More than 92% of the protein chains appear above the diagonal line, which means that BLCLUST PDFs are able to capture helpful information from flanking residues better than BETAN, so that the generally positive influence of flanking residues is better incorporated into the PDFs derived from BLCLUST compared to BETAN.

The advancement brought by this work is to articulate the nuanced influence of the flanking residues on the backbone conformation of the central residue, given the limited amount of structural data available. Exclusively measuring *I_n_*(*c*|*X_Z*), the average effect of flanking residues across the protein chain, gives some indication of the success of the methodology. For each of the 740 protein chains in BLC-NEW, the value for *I_n_*(*c*|*X_Z*) was measured using both BLCLUST and BETAN PDFs. The result, plotted in Figure 11, reveals that in BLCLUST PDFs the effect of the flanking residues are better elucidated than in BETAN—i.e., 92.3% of chains bear an improved *I_n_*(*c*|*X_Z*) with BLCLUST. Also, BLCLUST assigns negative *I_n_*(*c*|*X_Z*) to only 6.8% of the chains, compared to BETAN which assigns negative values to 33.8% of the chains. A negative value for *I_n_*(*c*|*X_Z*) suggests that, on average, the flanking residues do not assist in determining backbone conformation of a protein chain, an observation that is contrary to what is commonly assumed about local interactions in proteins. The much higher proportion of chains assigned negative *I_n_*(*c*|*X_Z*) by BETAN indicates that its triplet PDFs are not well-elucidated compared to BLCLUST triplet PDFs. Conclusively, the effect of the flanking residues on the conformation of the central residue backbone is more accurately defined by BLCLUST PDFs.

For further confirmation of the informatic superiority of the BLCLUST PDFs, two comprehensive threading tests were undertaken, the first involving local threading of 10-mer segments and the second involving whole chain threading using the CASP10 decoy set. For the first set of threading tests, a total of 5000 10-mers were picked randomly from BLC-NEW, and for each 10-mer 500 decoy conformations were assembled randomly from the PDBSEL data set. For the second set of threading tests, a total of 125 protein chains (with average length of 175 residues) were assembled from CASP10, along with an average of 367 high-resolution decoys per chain. The KBPs derived from BLCLUST and BETAN were used to score these sequence-conformation alignments, and the native score was ranked against the mass of decoy scores. For each native conformation, the difference in mutual information given by BLCLUST and BETAN was noted along with the resulting change in native score rank *r* in the threading test.

**Figure 12:**
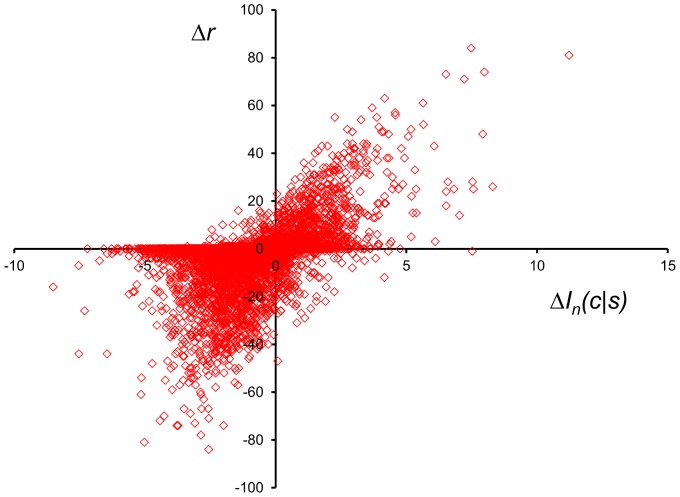
Comparison of BLCLUST and BETAN performance in folding recognition with local decoy threading. Threading trials were done to 5000 short segments randomly selected from the BLC-NEW data set, using two triplet sequence KBPs derived from BLCLUST and BETAN PDFs. Threading results are expressed in percentile rank *r*, while the native scores *I_n_*(*c*|*s*) were computed by Eq.(9). Each point in the plot represents one of 5000 short segments, whose coordinates are the difference in *r* of the native conformation in threading (x-axis) and the difference in *I_n_*(*c*|*s*) as given by the two KBPs. A positive Δ*I_n_*(*c*|*s*) means that BLCLUST assigns a higher mutual information score than BETAN. A positive Δ*r* means that native conformations are assigned lower (better) ranks by using BLCLUST KBPs than by BETAN KBPs. The strong correlation between the assignment of higher scores and the ability to detect native conformations is evidence of the superiority of BLCLUST PDFs over BETAN PDFs (See Table 3 for the details of the results of this threading test.).

The results for each 10-mer threading, summarized in Table 3 and plotted in Figure 12, confirms once again the solid correlation between an increase in mutual information and an improvement in performance as exemplified by a decrease in native score rank. For 70.6% of 10-mers, BLCLUST assigned a higher mutual information value than BETAN, resulting in a marked decrease in *r* for about 76.4% of the 10-mers. Aggregately, the mean mutual information increase is 0.11 nats while the mean decrease in native score percentile rank <*r*> is 3.23%. BLCLUST PDFs are significantly superior in recognizing native folds than BETAN PDFs.

**Table 3:** Threading results for KBPs using PDFs derived from BETAN and BLCLUST.

		BETAN triplet	BLCLUST triplet
**10-mer threading test**	*n* 10-mer^a^	5535	5535
	n decoys^b^	5000	5000
	<*r*>^c^	11.31	8.08
	<*I*-nat>^d^	0.265	0.372
	<*I*-dec>^e^	−0.347	−0.519
	*J* f	0.612	0.891
**CASP10 threading test**	n chains^g^	125	125
	n decoys^h^	367	367
	<*r*>^i^	11.66	9.81
	<*I*-nat>^d^	0.264	0.354
	<*I*-dec>^e^	0.058	0.013
	*J* f	0.206	0.341

a The number of 10-mer segments picked randomly from the data set BLC-NEW and subjected to threading test.

b The number of random decoys per chain.

c The percentile rank of the native conformation score amidst 5000 decoy conformation scores.

d The mean mutual information score (Eq.9) of the native conformation.

e The mean mutual information score computed for decoy conformations.

f The total divergence score, an information-theoretic quantity defined as <*I*-nat> - <*I*-dec>, which measures the mean gap between native scores and incorrect scores (see Ref. 23).).

g The number of chains in the CASP10 set.

h The average number of decoys per chain in the CASP10 set. The average chain length is 175 residues.

i The percentile rank of the native conformation score amidst the decoy conformation scores in the CASP10 set.

**Figure 13:**
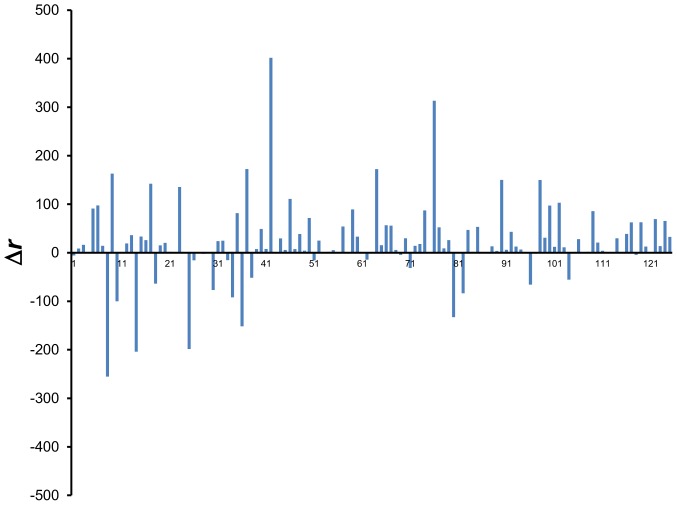
Comparison of BLCLUST and BETAN performance in fold recognition with CASP10 decoy threading. The improvement in percentile rank of the native conformation is shown for each of the 125 CASP10 chains in the data set. The horizontal axis marks each of the 125 protein chains, and the vertical axis is the difference in the percentile rank Δ*r* of the native conformation as determined by KBPs using BLCLUST and BETAN phi-psi maps. A positive Δ*r* means that KBPs using BLCLUST phi-psi maps see an improvement in discrimination (as measured by the percentile rank) compared to KBPs using BETAN phi-psi maps. Of the 125 chains, BLCLUST-based KBPs are able to assign equal or better rank to the native conformation of 80% of the CASP10 chains than BETAN-based KBPs (See Table 3 for the details of the results of this threading test.).

**Figure 14:**
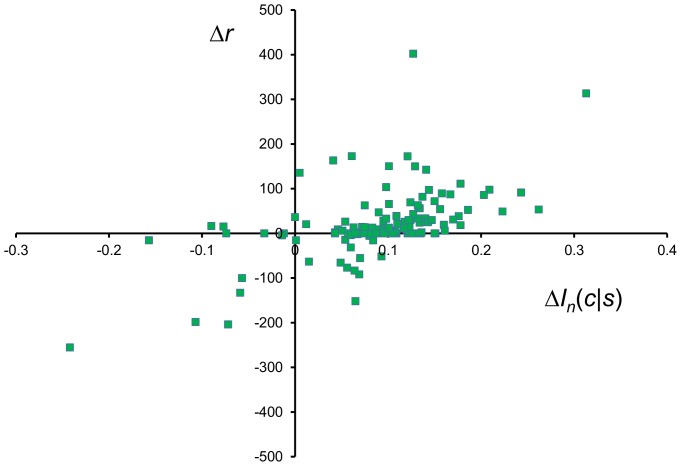
Comparison of BLCLUST and BETAN performance in fold recognition with CASP10 decoy threading. Threading trials were done to 125 protein chains in the CASP10 data set, using two triplet sequence KBPs derived from BLCLUST and BETAN PDFs. Threading results are expressed in percentile rank *r*, while the native scores *I_n_*(*c*|*s*) were computed by Eq.(9). Each point in the plot represents one of 125 chains, whose coordinates are the difference in *r* of the native conformation in threading (x-axis) and the difference in *I_n_*(*c*|*s*) as given by the two KBPs. A positive Δ*I_n_*(*c*|*s*) means that BLCLUST assigns a higher mutual information score than BETAN. A positive Δ*r* means that native conformations are assigned lower (better) ranks by using BLCLUST KBPs than by BETAN KBPs. The plot shows that most chains are assigned higher mutual information scores by BLCLUST KBPs, resulting in better threading discrimination as measured by the improvement in percentile rank of the native conformation. The strong correlation between the assignment of higher scores and the ability to detect native conformations is evidence of the superiority of BLCLUST PDFs over BETAN PDFs (See Table 3 for the details of the results of this threading test.).

The results for the whole-chain threading using CASP10 decoys are shown in Table 3 and plotted in Figures 13 and 14. Using triplet-sequence-dependent phi-psi maps derived in this work (BLCLUST) was able to assign equal or lower rank to native conformations of 80% of the 125 chains than the BETAN phi-psi maps. This is shown graphically in Figure 13, where the difference in percentile rank is shown for each of the 125 chains in CASP10. The mean percentile rank for native CASP10 conformations is 9.81% for KBPs that use BLCLUST phi-psi maps, significantly better than 11.66% when BETAN phi-psi maps are used instead (see Table 3 for details). Figure 14, which plots the mutual information score increase arising from the use of BLCLUST over BETAN phi-psi maps against the improvement gained in percentile rank of the native conformation, shows a similar pattern to Figure 12. Again, it is demonstrated here that KBPs that use BLCLUST phi-psi maps are able to increase the scores for native conformations, thereby improving discrimination amidst a challenging set of decoys in a fold recognition exercise.

These results are relevant to efforts that use knowledge-based structural propensity distributions for structure validation and model refinement. These procedures are in essence fold recognition exercises: a putative structure is scored by some energy or probabilistic function, and its viability is judged by how “normal” or “expected” the structure is by an implicit or explicit comparison with decoy or otherwise unnatural structures. Good PDFs (and their associated KBP) ought to score native conformations well, while also penalizing incorrect conformations by poor scores. Compared to BETAN PDFs, BLCLUST PDFs are shown here to assign higher mutual information (scores) to native conformations and also to more effectively discriminate against incorrect conformations. Structure validation and model refinement procedures should, in principle, benefit from information-optimized PDFs.

**Figure 15:**
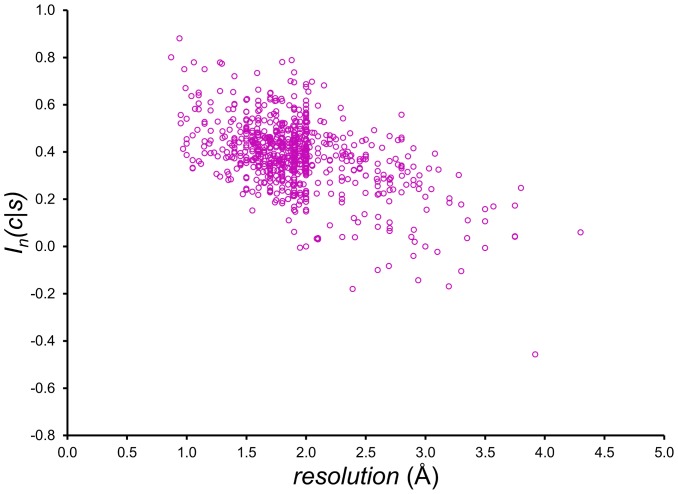
Dependence of mutual information score *I_n_*(*c*|*s*) on crystallographic resolution. For each of the 740 protein chains in the BLC-NEW data set, the score *I_n_*(*c*|*s*), derived from BLCLUST KBPs, is computed using Eq.(9) and plotted against the crystallographic resolution (in Ångstroms) of its experimental structure. A generalized correlation can be observed in this initial study. High-resolution structures are expected to contain phi-psi angles in the normal regions of the Ramachandran space, which are highly populated and should produce high mutual information scores. Conversely, lower resolution structures may contain a number of unnatural phi-psi angles that are penalized by the *I_n_*(*c*|*s*) function. This initial exploration points to the possibility of using triplet PDFs in structure validation and model refinement.

To begin to explore the viability of BLCLUST PDFs in structure validation and similar applications, BLCLUST KBPs were used to score the native conformations of 740 chains in BLC-NEW, a diverse collection of newly solved protein structures that are not homologous to any proteins in BLCLUST. In Figure 15, the resolution for each chain was plotted against its *I_n_*(*c*|*s*) score (Eq.9), which can be taken as a measure of the “normalness” of the phi-psi angle pairs of the experimental structure. The higher the *I_n_*(*c*|*s*) score, the more the phi-psi angle pairs conform, on average, to expected and highly populated values. A general correlation can be observed in Figure 15: specifically, low resolution crystal structures tend to have relatively lower *I_n_*(*c*|*s*) scores compared to higher resolution structures. This is because structures of low resolution will likely contain phi-psi angle pairs outside natural regions of the Ramachandran space, which the *I_n_*(*c*|*s*) function is able to detect and penalize. This initial observation supports the hypothesis that these phi-psi maps are potentially useful in structure validation and model refinement. Confirmation of this hypothesis by more extensive measurements, and formalizing the use of *I_n_*(*c*|*s*) as a structure validation parameter, are among the future directions arising from this work.

### F. Concluding Remarks

Deriving sequence-dependent structural propensities from structure data is straightforward in principle—a database is assembled from which frequencies are extracted and converted into probability distribution functions (PDFs). The number of adjustable variables, however, presents a challenge if the goal is to derive the best PDFs from given data. Information theory provides an elegant and powerful way to build PDFs that optimize all aspects of the process—from determining the most sensible descriptors for sequence and conformation to improving the way probabilities are derived from frequency counts. The work described here reveals a straightforward procedure that results in superior PDFs that maximize the extraction of structural information from empirical data.

The specific goal of this work is to build the most accurate phi-psi dihedral angle probability distribution functions (PDFs) for all 20 single amino acids and all 8000 triplet sequences from high-resolution crystal data. The fundamental question of how to extract the most accurate functions given empirical data has prompted the information-theoretic analysis explored here. The outcome of this analysis convincingly points to the maximization of mutual information estimates *I_E_*(*C*,*S*) as the correct objective function in the optimization of PDFs. The advantages of using mutual information are known [23], [24], and once again confirmed here—that the resulting knowledge-based potentials perform best in fold recognition tests, and that parameter optimization can be achieved by looking only at the “energies” of native sequence-structure alignments, bypassing the costly need to explore the “energy gap” between the correct structural state and a large set of decoys. Yet another advantage has been demonstrated in this work: empirical PDFs that maximize *I_E_*(*C*,*S*) are the best approximation for the underlying true probabilities given limited data.

The triplet sequence PDFs have been derived from all high-resolution crystal structures in the Protein Data Bank (PDB), including redundant and near-identical sequences. A procedure was devised to weight the contribution of each chain inversely with the size of the cluster of similar sequences in which it belongs. Frequency counts were made using the dynamic radius approach, which extracts more information than standard static binning common in the literature. The resolution, an important adjustable parameter, was also optimized. The performance of these PDFs in comprehensive threading tests is superior to a recent set derived by Betancourt [16], and points to a greater ability to elucidate the nuanced influence of the flanking residues on the backbone conformation of the central amino acid. Such functions may prove useful as tools for structure validation, as components of knowledge-based potentials (KBPs), and as clues that may lead to understanding complex molecular interactions and the very nature of protein folding.

The Information Maximization Device (IMD) encapsulates the simple computational approach to optimizing PDFs as well as KBPs that rely on accurate PDFs. The ingredients are the following: two distinct structural data sets and a procedure to turn sparse frequencies into well-defined probabilities. One data set, the training data set, is used to derive probability estimates; another data set, the testing data set, is used to compute each term of the summation of the IMD. The composition of the training data set has virtually no restrictions; its effectiveness will be determined ultimately by the *I_E_*(*C*,*S*) resulting from the PDFs. The composition of the testing data set, however, is crucial, because it ought to reflect the range of structures that the PDF aims to describe as well as the sequence-structure space to which the associated KBPs will be applied. The two data sets can overlap, but a valid jackknife procedure is necessary when computing each term in the IMD summation. It should be noted that the training data set, along with the other ingredient of the IMD, the probability estimation procedure, can be integrated into the procedure as variables to be optimized as well. Indeed, in the work described here, an expanded data set, BLCLUST, was proven to be superior as the training set—both in terms of *I_E_*(*C*,*S*) and performance in threading—to a more limited, non-redundant data set, PDBSEL. The probability estimation procedure may have a number of parameters, all of which can be optimized in a similar fashion.

Beyond providing the most informative single amino acid and triplet phi-psi maps to date, this work has wider implications. Close analysis reveals that these plots can still benefit from the continued increase in the size of the PDB. As expected, triplet sequence maps will become increasingly accurate with more observations. But even the 20 amino acid maps, with the abundance of occurrences in the database, can benefit from periodic reevaluation. In addition, the simple mechanism of IMD can be employed to explore various other sequence-dependent conformations. Lastly, the training data set can be expanded to include high-resolution structures of redundant and near-identical sequences—in fact, there is latent information in the minute conformational variability that occurs among related sequences, so that including them in statistical analysis and model building is ultimately beneficial.
